# Endocytic Crosstalk: Cavins, Caveolins, and Caveolae Regulate Clathrin-Independent Endocytosis

**DOI:** 10.1371/journal.pbio.1001832

**Published:** 2014-04-08

**Authors:** Natasha Chaudhary, Guillermo A. Gomez, Mark T. Howes, Harriet P. Lo, Kerrie-Ann McMahon, James A. Rae, Nicole L. Schieber, Michelle M. Hill, Katharina Gaus, Alpha S. Yap, Robert G. Parton

**Affiliations:** 1 The University of Queensland, Institute for Molecular Bioscience, Queensland, Australia; 2 The University of Queensland, Diamantina Institute, Queensland, Australia; 3 The University of New South Wales, Centre for Vascular Research and Australian Centre for Nanomedicine, New South Wales, Australia; 4 Centre for Microscopy and Microanalysis, Queensland, Australia; UT Southwestern Medical Center, United States of America

## Abstract

Several studies have suggested crosstalk between different clathrin-independent endocytic pathways. However, the molecular mechanisms and functional relevance of these interactions are unclear. Caveolins and cavins are crucial components of caveolae, specialized microdomains that also constitute an endocytic route. Here we show that specific caveolar proteins are independently acting negative regulators of clathrin-independent endocytosis. Cavin-1 and Cavin-3, but not Cavin-2 or Cavin-4, are potent inhibitors of the clathrin-independent carriers/GPI-AP enriched early endosomal compartment (CLIC/GEEC) endocytic pathway, in a process independent of caveola formation. Caveolin-1 (CAV1) and CAV3 also inhibit the CLIC/GEEC pathway upon over-expression. Expression of caveolar protein leads to reduction in formation of early CLIC/GEEC carriers, as detected by quantitative electron microscopy analysis. Furthermore, the CLIC/GEEC pathway is upregulated in cells lacking CAV1/Cavin-1 or with reduced expression of Cavin-1 and Cavin-3. Inhibition by caveolins can be mimicked by the isolated caveolin scaffolding domain and is associated with perturbed diffusion of lipid microdomain components, as revealed by fluorescence recovery after photobleaching (FRAP) studies. In the absence of cavins (and caveolae) CAV1 is itself endocytosed preferentially through the CLIC/GEEC pathway, but the pathway loses polarization and sorting attributes with consequences for membrane dynamics and endocytic polarization in migrating cells and adult muscle tissue. We also found that noncaveolar Cavin-1 can act as a modulator for the activity of the key regulator of the CLIC/GEEC pathway, Cdc42. This work provides new insights into the regulation of noncaveolar clathrin-independent endocytosis by specific caveolar proteins, illustrating multiple levels of crosstalk between these pathways. We show for the first time a role for specific cavins in regulating the CLIC/GEEC pathway, provide a new tool to study this pathway, identify caveola-independent functions of the cavins and propose a novel mechanism for inhibition of the CLIC/GEEC pathway by caveolin.

## Abstract 2

Caveolar proteins and caveolae negatively regulate a second clathrin-independent endocytic CLIC/GEEC pathway; caveolin-1 affects membrane diffusion properties of raft-associated CLIC cargo, and the scaffolding domain of caveolin-1 is required and sufficient for endocytic inhibition.

## Author Summary

Endocytosis is the process that allows cells to take up molecules from the environment. Several endocytic pathways exist in mammalian cells. While the best understood endocytic pathway uses clathrin, recent years have seen a great increase in our understanding of clathrin-independent endocytic pathways. Here we characterize the crosstalk between caveolae, flask-shaped specialized microdomains present at the plasma membrane, and a second clathrin-independent pathway, the CLIC/GEEC Cdc42-regulated endocytic pathway. These pathways are segregated in migrating cells with caveolae at the rear and CLIC/GEEC endocytosis at the leading edge. Here we find that specific caveolar proteins, caveolins and cavins, can also negatively regulate the CLIC/GEEC pathway. With the help of several techniques, including quantitative electron microscopy analysis and real-time live-cell imaging, we demonstrate that expression of caveolar proteins affects early carrier formation, causes cellular lipid changes, and changes the activity of the key regulator of the CLIC/GEEC pathway, Cdc42. The functional consequences of loss of caveolar proteins on the CLIC/GEEC pathway included inhibition of polarized cell migration and increased endocytosis in tissue explants.

## Introduction

Endocytosis encompasses a number of distinct internalization pathways with clathrin-mediated endocytosis (CME) of receptors and their bound ligands being the best understood [1]–[7]. Caveolae, cup-shaped invaginations of the cell surface, have also received much attention as endocytic vehicles [8]–[10]. However, the contribution to cellular endocytic uptake may vary greatly between cell types and conditions, reflecting striking tissue-specific distribution and, presumably, functions of caveolae [11]–[13]. Caveola biogenesis involves the core structural membrane proteins CAV1 and muscle-specific CAV3, which are essential for caveola formation. A coat protein complex, consisting of cavin family proteins, Cavin-1, Cavin-2, and Cavin-3, and muscle-specific Cavin-4 has been shown to associate with caveolae at the plasma membrane (PM) [14]–[19], and together with the GTPase dynamin, the ATPase EHD2, and pacsin2, regulates caveola formation and dynamics [20],[21]. Studies of caveolae have traditionally followed expressed caveolins as markers of caveolae, but it is now apparent that caveolins depend on cavins and associated proteins for association with, and formation of, caveolae. Excess non caveolar caveolin can be rapidly internalized and degraded, for example when caveolin is over-expressed or upon down-regulation of cavin proteins [18],[22]. In contrast, caveolae generally bud off from the PM and recycle back to the surface, transitioning through classical Rab5-positive early endosomes. While caveolar endocytosis may not be a high capacity route in most cell types, the cycle of endocytosis and recycling is required for maintaining a constant caveolar density at the cell surface [13],[23],[24].

Caveolin-independent, clathrin-independent (CI) endocytic routes have, until recently, escaped extensive characterization because of the lack of specific cargo or regulators limiting the available biochemical or molecular tools to study their unique features. Hence, insights into clathrin-independent endocytosis (CIE) have been derived from combining general markers, such as fluid phase probes or membrane markers [25], with cellular systems that lack or have been manipulated to inhibit the classical clathrin and caveolin routes. More recently, a number of endogenous cargo molecules trafficking by distinct, constitutive CI pathways have been identified, including glycosylphosphatidylinositol-anchored proteins (GPI-APs) [26], the interleukin-2 receptor [27], and the major histocompatibility complex I [28]. These pathways differ based upon their dependence on dynamin function and reliance on small GTPases, namely Cdc42, RhoA, or Arf6, for cargo internalization [1],[4],[5].

Here, we have focused on the Cdc42-regulated, GPI-AP-positive CLIC/GEEC (clathrin-independent carriers/GPI-AP enriched early endosomal compartment) pathway, which constitutes a high capacity route for bulk fluid intake in fibroblasts [29]. Previous work has shown the unique, tubular morphology of the primary carriers (CLICs) in this pathway [23] and defined the early stages in the formation of these carriers: clustering of CLIC cargo, such as GPI-APs, within Cdc42-positive regions of the plasma membrane and requirement of local actin polymerization during formation of the carriers [26],[30],[31]. Using multiple fluid phase markers and pulse-chase experiments, it was shown that after CLICs are formed they bud rapidly, within 15 seconds, from the PM and acquire Rab5 and EEA-1, maturing into the GEEC stage, before fusion with early endosomes and mixing with cargo from the CME pathway, such as transferrin (Tfn) [23],[32]. Other recently revealed key regulators of the CLIC/GEEC pathway include a regulator of secretory traffic, Arf1, and GRAF1 (GTPase regulator associated with focal adhesion kinase-1) [33],[34].

CLIC/GEEC endocytosis and caveolae share several similar properties, including the involvement of actin machinery, an important role for free cholesterol, and the action of specific protein/lipids residing in sphingolipid-rich membrane rafts [25],[30],[35]–[37]. However, the two pathways show striking differences in migrating cells: CLIC-mediated endocytosis occurs at the leading edge of the cell while caveolae are localized to the rear [29],[38]. Interestingly, over-expression of CAV1, in cells either lacking CAV1 or containing endogenous CAV1, has been shown to inhibit dynamin-independent internalization of cholera toxin subunit b (CTxB) and fluid phase markers [23],[39]. Whether this has physiological relevance is not yet known.

In this study we have refined and validated our systems that follow CLIC endocytosis and used them to study potential crosstalk with caveolae. Using internalized antibodies to the hyaluronan receptor, CD44, as a CLIC-specific marker [29], our studies reveal complex crosstalk between the two membrane systems at multiple levels.

## Results

### CD44 as a Marker for CLIC/GEEC Endocytosis

**Figure 1:**
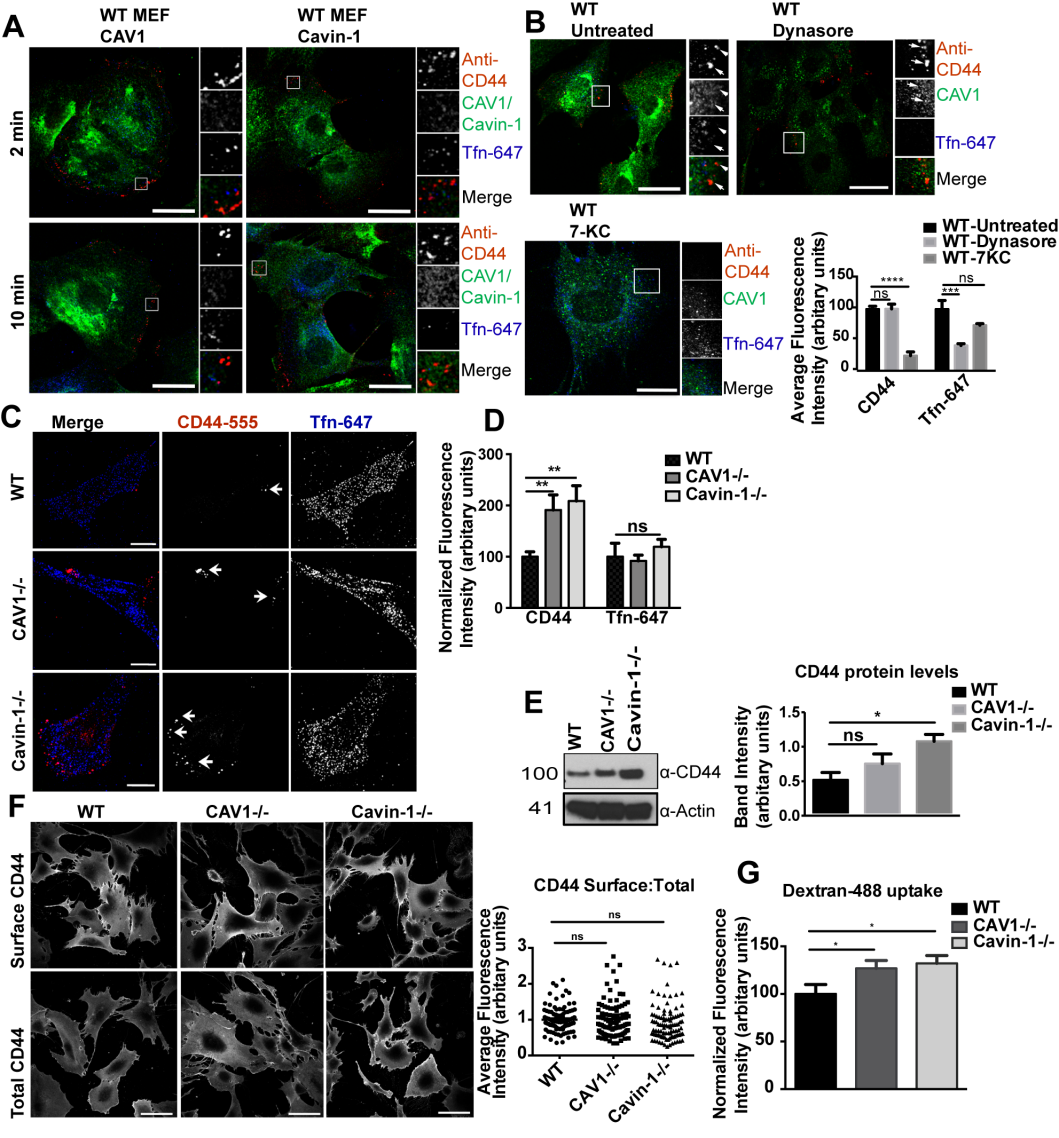
CD44 as a marker of CLIC/GEEC endocytosis. (A) WT MEFs were incubated with anti-CD44 mAb and Tfn-647 for 2 min and 10 min at 37°C. Cells were placed on ice and acid washed before fixation. Internalized anti-CD44 mAb was labeled with anti-mouse Alexa Fluor-555 (AF-555) secondary antibody. For labeling endogenous CAV1 and Cavin-1, respective primary antibodies were used, followed by respective secondary AF antibodies. (B) WT MEFs were treated with 60 µM dynasore and 30 µM 7-KC respectively or left untreated (control) for 30 min at 37°C prior to uptake of endocytic markers as mentioned in (A). The bar graph represents the quantitation of internalized endocytic markers. (C) Internalization assay with anti-CD44 mAb and Tfn-647 was performed in WT, CAV1−/−, and Cavin-1−/− MEFs for 2 min at 37°C as mentioned in (A). Arrows indicate CD44-labeled puncta. (D) 40–50 cells from (C) were quantified for normalized fluorescent intensity of endocytic markers. (E) Whole cell lysates from WT, CAV1−/−, and Cavin-1−/−MEFs were immunoblotted for CD44. A representative immunoblot is shown in which actin was used as a loading control. The bar graph represents quantitation of CD44 protein levels calculated by measuring band intensities, by densitometry, in WT, CAV1−/−, and Cavin-1−/− MEFs from three independent experiments. (F) Both nonpermeabilized and permeabilized WT, CAV1−/−, and Cavin-1−/− MEFs were labeled with primary anti-CD44 mAb followed by secondary AF-488 labeling and Phalloidin-AF-596 (F-actin, not shown) as an internal control. 50–60 cells from each condition and cell type were quantified for average fluorescent intensity ratio of CD44 and F-actin (CD44:F-Actin). The graph represents ratio of CD44:F-actin values obtained in nonpermeabilized (surface) cells normalized by the corresponding value obtained in permeabilized (total) cells. (G) Internalization assay with Dex-488 was performed in WT, CAV1−/−, and Cavin-1−/− MEFs for 5 min at 37°C. 40–50 cells from each cell type were quantified for normalized fluorescent intensity of internalized Dex-488. In (F) data represent mean ± SEM of data pooled from three independent experiments, and statistical significance was calculated by one-way ANOVA analysis. In (B, D, E, G) data represent mean ± SEM of three independent experiments. *p<0.02,**p<0.01,***p<0.001,****p<0.0001 (two-tailed t-test). Scale bar: 10 µm.

Various markers have been used to follow CIE pathways but CD44 has emerged as a highly specific cargo of the CLIC/GEEC pathway [29],[40]. We therefore first optimized and validated the conditions for quantitatively studying CIE using antibodies against CD44 as a marker. An anti-CD44 monoclonal antibody (mAb) was added together with fluorescent transferrin (Tfn-647) to cells for 2 and 10 min at 37°C. Prior to fixation, cells were placed on ice and acid stripped to remove any residual surface label, and internalized CD44 mAb was labeled with fluorescently tagged secondary antibody. For quantitative analysis of internalization, the fluorescence intensity of the internalized markers (over the entire cell) was normalized against the average fluorescence intensity of the internalized markers in control samples. By the following criteria, this procedure allowed us to use CD44 as a specific marker of the CLIC/GEEC pathway: (1) Anti-CD44 mAb internalization was distinct from uptake via clathrin coated pits, labeled with Tfn-647, and did not colocalize with caveolae, indicated by probing for endogenous caveolar proteins, CAV1 and Cavin-1 (Figure 1A). (2) CD44 mAb uptake was dynamin-independent, as determined by the use of the small molecule inhibitor dynasore. Under our experimental conditions, dynasore treatment inhibited Tfn-647 uptake but had no effect on CD44 mAb internalization (Figure 1B) [29]. (3) Uptake of CD44 mAb was completely inhibited by 7-ketocholesterol (7-KC) treatment, shown in previous studies to result in reduced membrane order [41], while Tfn-647 uptake was not significantly affected at this concentration (Figure 1B). These results confirmed that in our system anti-CD44 antibody was internalized via the cholesterol-dependent, dynamin-independent CLIC/GEEC pathway [23],[30] (Figure 1B). Internalized anti-CD44 mAb therefore represents a specific marker of the CLIC/GEEC pathway that does not require a low temperature prebinding step and does not affect the kinetics or the magnitude of the pathway being studied [29]. Specific internalization of anti-CD44 mAb was confirmed by using a control antibody (anti-GFP mAb) at the same concentration in wild type mouse embryonic fibroblast cells (WT MEFs) and with anti-CD44 mAb in COS-7 cells, which lack CD44 receptor on the cell surface [42]. Under both conditions, no internalized antibody could be detected after 2 min at 37°C whereas COS-7 cells expressing CD44-GFP showed specific uptake of the anti-CD44 mAb (Figure S1A,B). Additionally, using live cell imaging, we also observed colocalization between CD44-GFP and the fluid phase marker (Dex-647), suggesting that CD44-GFP is internalized via the CLIC pathway (Figure S2, Movie S1). These internalization conditions were subsequently used to investigate the crosstalk between the caveolar and the CLIC/GEEC endocytic systems.

### The CLIC/GEEC Endocytic Pathway is Up-regulated in CAV1 and Cavin-1 Null Cells

Transient CAV1 over-expression, which leads to caveola formation in cells that express Cavin-1, has been shown to inhibit CIE [23],[39],[43]–[46]. To investigate whether this reflects a general role of caveolar proteins in the CLIC/GEEC pathway, we used CAV1 null (CAV1−/−) and Cavin-1 null (Cavin-1−/−) MEFs (note these cells also have lower Cavin-1 and CAV1 protein levels, respectively, and so do not allow discrimination of specific effects of loss of either protein (Figure S3A) [18]). We compared the constitutive uptake of CD44 mAb and Tfn-647 in WT, CAV1−/−, and Cavin-1−/− MEFs after 2 min at 37°C; at this early time point of internalization, peripheral labeling for CD44 mAb was observed, consistent with a previous study [29] (Figure 1C). A significant 2-fold increase in anti-CD44 mAb fluorescence, as a measure of CLIC internalization, was observed in CAV1−/− and Cavin-1−/− MEFs in comparison with WT MEFs (Figure 1D). However, there was no significant effect on Tfn-647 uptake, indicating that CME was not affected (Figure 1C,D). Additionally, CD44 mAb and Tfn-647 uptake was also performed in CAV1−/− MEFs expressing Cavin-1-specific siRNA to characterize the functional consequences, if any, of loss of Cavin-1 in CAV1−/− MEFs. No significant difference was observed in either CD44 mAb or Tfn-647 uptake between Cavin-1 siRNA (80% knock down) and control siRNA-transfected CAV1−/− cells (Figure S3B). We also examined whether the increase in anti-CD44 mAb internalization was related to altered CD44 protein expression levels in WT, CAV1−/−, and Cavin-1−/− MEFs. Western blot analysis showed no statistically significant increase in the expression level of CD44 in CAV1−/− MEFs but an increase in Cavin-1−/− MEFs when compared with WT cells (Figure 1E). However, quantitative immunofluorescence (IF) analysis showed no significant difference in the ratio of surface/total levels of anti-CD44 mAb labeling between WT, CAV1−/−, and Cavin-1−/− MEFs (Figure 1F).

Altogether, these results suggest that the CLIC/GEEC pathway may be upregulated in the absence of caveolar proteins. We therefore investigated the effect of loss of caveolar proteins on internalization of the fluid phase marker fluorescent dextran (Dex-488), which is partially internalized through the CLIC/GEEC pathway [23],[26]. Dex-488 was internalized at 37°C for 5 min to provide sufficient signal-to-noise ratio. Similar to CD44 mAb uptake, a significant increase in Dex-488 internalization was observed in CAV1−/− and Cavin-1−/− MEFs in comparison with WT MEFs (Figure 1G). Together, these data suggest that lack of caveolae, through two distinct genetic manipulations, causes upregulation of the CLIC/GEEC pathway. Thus, we next investigated the role of specific caveolar proteins in this regulation.

### Caveolae, Caveolin-1, and Cavin-1 Inhibit Uptake via the CLIC/GEEC pathway by Independent Mechanisms

**Figure 2:**
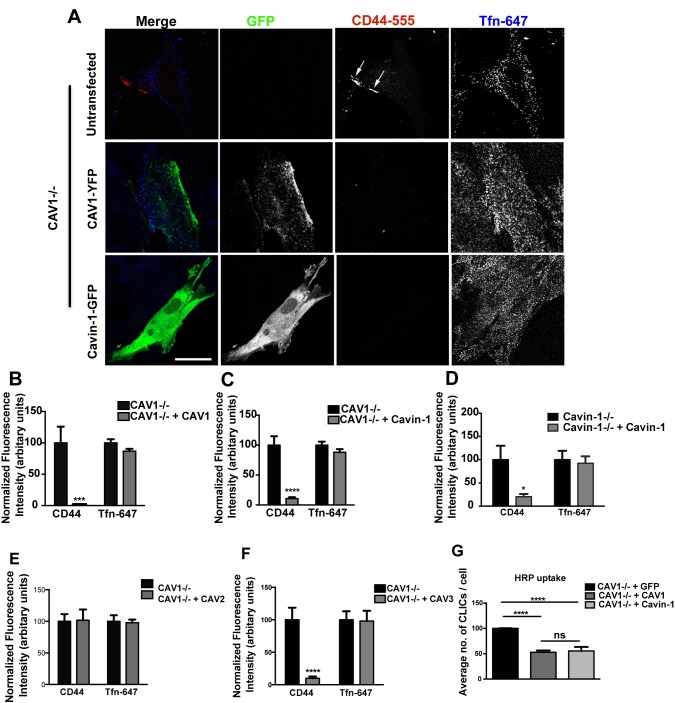
Caveolar proteins specifically down-regulate CLIC endocytosis. (A) In CAV1−/− MEFs transiently expressing CAV1-YFP and Cavin-1-GFP respectively, internalization assay was performed with anti-CD44 mAb and Tfn-647 for 2 min at 37°C. Cells were acid washed prior to fixation, and internalized anti-CD44 mAb was labeled with AF-555 secondary antibody. Arrows indicate CD44-labeled puncta. (B,C) 40–50 cells of each transfection from (A) were quantified for normalized fluorescent intensity of endocytic markers. (D) Cavin-1−/− MEFs were transiently transfected with Cavin-1-GFP, and uptake and quantification of internalized anti-CD44 mAb and Tfn-647 were performed as mentioned in (A,B). (E,F) CAV1−/− MEFs were transiently transfected with Myc-CAV2 and RFP-CAV3 respectively. Uptake and quantification of internalized anti-CD44 mAb and Tfn-647 were performed as mentioned in (B). (G) In CAV1−/− MEFs transiently expressing GFP vector, CAV1-YFP, and Cavin-1-GFP respectively, constitutive internalization of HRP (10 µg/ml) was performed for 2 min at 37°C followed by DAB reaction on live cells. Cells were fixed, embedded, and sectioned. Quantification of HRP-labeled CLICs per cell, using 16-20 cells from each transfection, is shown. For more images representing experimental variation obtained for uptake analysis, performed in untransfected and CAV1-YFP/Cavin-1-GFP-expressing CAV1−/− MEFs, see supporting information Figure S12, Figure S13, and Figure S14. In (B–G) data represent mean ± SEM of three independent experiments. *p<0.05,***p<0.0005,****p<0.0001 (two-tailed t-test). Scale bar: 10 µm.

We restored the levels of CAV1 and Cavin-1 in CAV1−/− MEFs by expressing the fluorescent protein (YFP/GFP) tagged constructs, and then compared uptake of the CD44 mAb (2 min at 37°C) between transfected and untransfected cells (Figure 2A; Figure S4). We observed 30–40% transfection efficiency for almost all transient transfections and only low transgene-expressing cells were analyzed. CD44 mAb endocytosis was drastically reduced in CAV1-expressing cells (Figure 2B). More surprisingly Cavin-1 expressing CAV1−/− MEFs also showed a dramatic decrease in CD44 mAb endocytosis (Figure 2C). As CAV1−/− cells lack morphological caveolae [23] this strongly suggests that Cavin-1 inhibits CLIC endocytosis independent of CAV1 and caveolae. Additionally, exogenous expression of Cavin-1 in Cavin-1−/− MEFs was also observed to cause a significant decrease in CD44 mAb uptake (Figure 2D). Neither CAV1 nor Cavin-1 expression affected Tfn-647 uptake. Compared with untransfected cells, CAV1 over-expression resulted in 95±0.3% inhibition of CD44 mAb endocytosis, while Cavin-1 caused a 70±2.2% inhibition (Figure 2B,C). Since loss of caveolar proteins resulted in an increase in Dex-488 uptake, we also investigated the effects of reconstituted CAV1 and Cavin-1 expression in CAV1−/− MEFs on Dex-488 uptake (5 min at 37°C). Similar to CD44 mAb uptake, expression of either CAV1 or Cavin-1 resulted in a significant decrease in Dex-488 uptake (Figure S5A,B; 40±7% inhibition in CAV1-expressing cells; 44±6% inhibition in Cavin-1 expressing cells) although not as high inhibition as observed with the specific CLIC marker, CD44 mAb. We also characterized the effect of other members of the caveolin family, CAV2 and CAV3, on the CLIC/GEEC pathway. The ectopic expression of CAV3, but not CAV2, inhibited CD44 mAb uptake in CAV1−/− MEFs, while Tfn-647 uptake was not affected by expression of either of the proteins (Figure 2E,F). To test whether inhibition of the CLIC/GEEC pathway activity upon expression of caveolar proteins in our system (CAV1−/− MEFs) is due to inhibition of CLIC/GEEC carrier formation, we used electron microscopy (EM). CAV1−/− MEFs were transiently transfected with CAV1-YFP, Cavin-1-GFP, and with GFP alone respectively and then the fluid phase marker horseradish peroxidase (HRP) was added for 2 min at 37°C to label the putative CLIC/GEEC carriers [23],[29]. In comparison with cells expressing GFP alone, a significant decrease was observed in the number of CLICs in CAV1 and Cavin-1 transfected cells (GFP: 100%; CAV1: 54±4% decrease; Cavin-1: 55±8% decrease; mean ± SEM), indicating that expression of caveolar proteins can limit the formation of carriers of the CLIC/GEEC pathway (Figure 2G).

**Figure 3:**
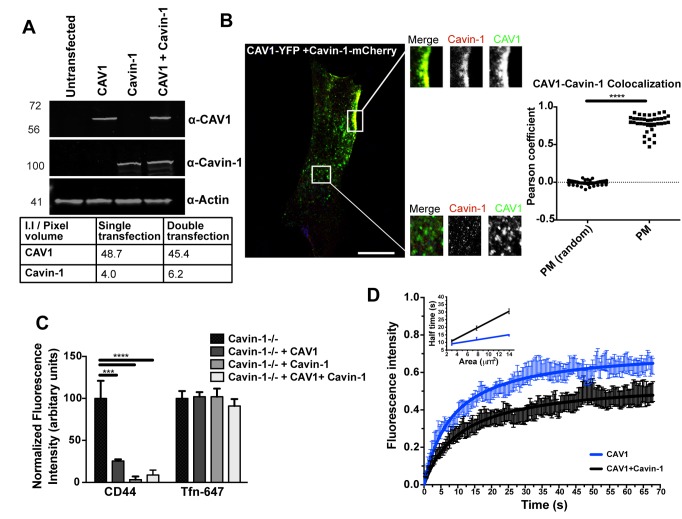
Caveolae generation down-regulates CLIC endocytosis. (A) Cell lysates from untransfected Cavin-1−/− MEFs or Cavin-1−/− MEFs transiently transfected with CAV1-YFP or Cavin-1-mCherry or co-transfected with CAV1-YFP and Cavin-1-mCherry were immunoblotted for CAV1 and Cavin-1. Actin was used as loading control. For quantification, the integrated intensity of protein bands was calculated. (B) Representative image of cell from (A) showing colocalization between CAV1 and Cavin-1 at different subcellular locations. Insets depict CAV1, Cavin-1 and merged labeling at the plasma membrane and cytosolic regions, marked by the square. 40 cells from (A) were subjected to Pearson correlation analysis, and a representative graph is shown. (C) Cells from (A), growing on coverslips, were subjected to internalization assay with anti-CD44 mAb and Tfn-647 for 2 min at 37°C. Cells were acid washed prior to fixation and internalized anti-CD44 mAb was labeled with AF-555 secondary antibody. 40–50 cells were quantified for normalized fluorescent intensity of endocytic markers. (D) FRAP analysis for CAV1 diffusion at the plasma membrane of Cavin-1−/− MEFs expressing CAV1-YFP alone or co-expressing CAV1-YFP and Cavin-1-mCherry. Fluorescence recovery curve and lateral diffusion analysis (inset) from 15 cells (mean ± SD) are shown. In (B) data represent mean ± SEM of data pooled from three independent experiments and in (C) data represent mean ± SEM of three independent experiments. ***p<0.0005,****p<0.0001 (two-tailed t-test). Scale bar: 10 µm.

These results suggested that both caveolins (CAV1 and CAV3) and Cavin-1 are capable of negatively regulating the CLIC/GEEC pathway. This raised the question of whether inhibition is caused exclusively by free caveolar proteins, independent of caveolar formation. We addressed this question by testing whether inhibition of the CLIC/GEEC pathway occurred when levels of caveolin and cavin were balanced, as indicated by their colocalization and by the immobilization of CAV1 within caveolae. Cavin-1−/− MEFs were transfected either with CAV1-YFP or with Cavin-1-mCherry alone or with both constructs simultaneously. Both CAV1 and Cavin-1 proteins were expressed in similar amounts in single and double transfections, as confirmed by quantitative Western blot analysis (Figure 3A). Moreover, confocal images showed negligible levels of cytosolic Cavin-1 present in CAV1 and Cavin-1 co-expressing cells, and quantitative colocalization analysis showed a significantly higher degree of colocalization between CAV1 and Cavin-1 at the PM in comparison with analysis of randomized pixels for the same regions (Pearson coefficient, PM: 0.80±0.02; random: −0.007±0.005; mean ± SEM, n = 40 cells, p<0.0001) (Figure 3B). In the same set of cells, anti-CD44 mAb and Tfn-647 uptake studies were performed. A significant decrease in CD44 mAb endocytosis was observed in cells co-transfected with CAV1 and Cavin-1, whereas Tfn-647 uptake was not affected (Figure 3C). To gain a quantitative estimation of free caveolar proteins present in our system upon co-expression of CAV1 and Cavin-1, we made use of fluorescence recovery after photobleaching (FRAP) to analyze the mobility and diffusion properties of CAV1. Cavin-1−/− MEFs were transiently transfected with CAV1-YFP or co-transfected with CAV1-YFP and Cavin-1-mCherry respectively. After photobleaching a defined region of interest (ROI) at the PM, we compared the rate of fluorescence recovery of CAV1 between the single and co-transfected cells. A significant decrease in the mobile fraction (CAV1: 0.82±0.03; CAV1+Cavin-1: 0.58±0.05, p<0.05) and lateral diffusion (CAV1: 0.13±0.08; CAV1+Cavin-1: 0.039±0.002; mean ± SD, n = 15 cells, p<0.001, see Text S1) of CAV1 was observed when co-expressed with Cavin-1, in comparison with expression alone (Figure 3D), suggesting that most of the CAV1 in the presence of Cavin-1 had been immobilized through the formation of caveolae. Taken together, these results suggest that the caveolar proteins, independent of caveolae, can inhibit CLIC endocytosis, but in addition, when incorporated into caveolae, there is also potent inhibition of CLIC endocytosis.

### Cavins 1 and 3, but Not 2 and 4, Inhibit the CLIC/GEEC Pathway

**Figure 4:**
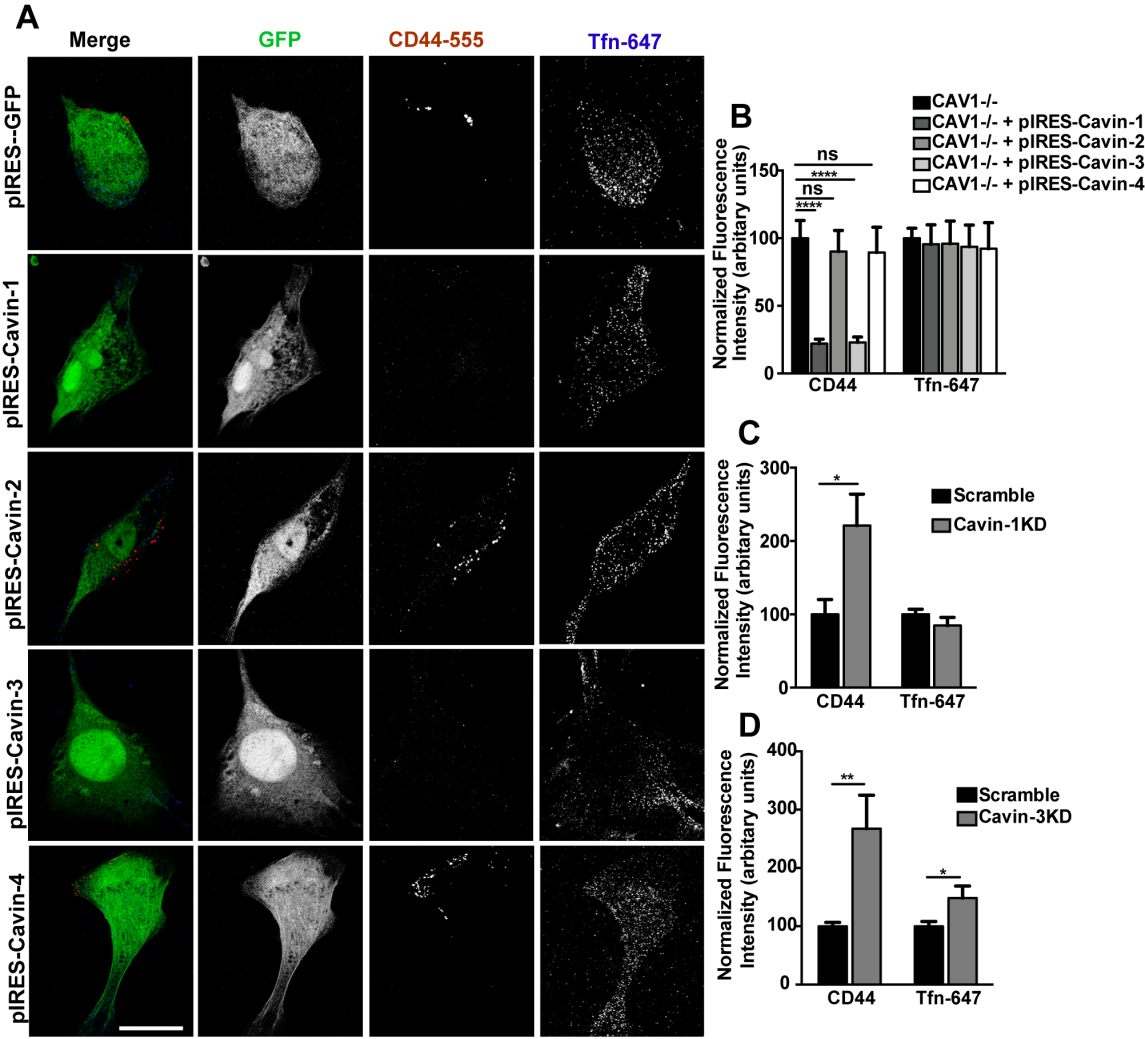
Cavin-1 and Cavin-3, but not Cavin-2 and Cavin-4 negatively regulate CLIC endocytosis. (A) CAV1−/− MEFs were transiently transfected with pIRES-GFP, pIRES-Cavin-1, pIRES-Cavin-2, pIRES-Cavin-3, and pIRES-Cavin-4 respectively and internalization assay was performed with anti-CD44 mAb and Tfn-647 for 2 min at 37°C. Cells were acid washed prior to fixation and internalized anti-CD44 mAb was labeled with AF-555 secondary antibody. (B) 40–50 cells from each transfection were quantified for normalized fluorescent intensity of endocytic markers. (C,D) 3T3-L1 cells were transiently transfected with siRNA directed specifically to Cavin-1 and Cavin-3 respectively. Uptake and quantification of internalized marker were performed as mentioned in (A,B). In (B–D) data represent mean ± SEM of three independent experiments. *p<0.05,**p<0.005,****p<0.0001 (two-tailed t-test). Scale bar: 10 µm.

To further investigate the unexpected regulatory role of cavins in CLIC/GEEC endocytosis, we analyzed the effects of ectopic expression of each of the four members of the cavin family (Figure S6A) on CD44 mAb internalization. In CAV1−/− MEFs, moderate over-expression of Cavin-1 and Cavin-3 caused a significant decrease in CD44 mAb endocytosis, whereas in Cavin-2 and Cavin-4-expressing cells no effect on CD44 mAb endocytosis was observed (Figure 4A). Expression of both Cavin-1 and Cavin-3 resulted in significant inhibition (Cavin-1: 77.9±3.3% decrease; Cavin-3: 76.9±3.9% decrease; mean ± SEM) of CD44 mAb endocytosis when compared with control cells (100±13%; Figure 4B). These results reaffirmed the noncaveolar role of Cavin-1 as a negative regulator of CLIC/GEEC endocytosis. Furthermore, Cavin-3 was also observed to have a CAV1- and caveola-independent regulatory role in CLIC/GEEC endocytosis.

We next assessed the effect of reducing cavin protein levels on the CLIC/GEEC pathway using a siRNA directed approach in 3T3-L1 fibroblasts, as we obtained more efficient knock down in this cell type compared with MEFs. Reduction of Cavin-1 levels (by approximately 80%) and Cavin-3 levels (by approximately 50%) resulted in an increase in CD44 mAb uptake in comparison with control siRNA-treated cells (Figure 4C,D; Figure S6B). Reduction of Cavin-3 levels also led to an increase in Tfn-647 uptake while reduction in Cavin-1 did not, suggesting Cavin-3 might influence CME.

### The Scaffolding Domain of CAV1 is Essential and Sufficient for Inhibition of the CLIC/GEEC pathway

**Figure 5:**
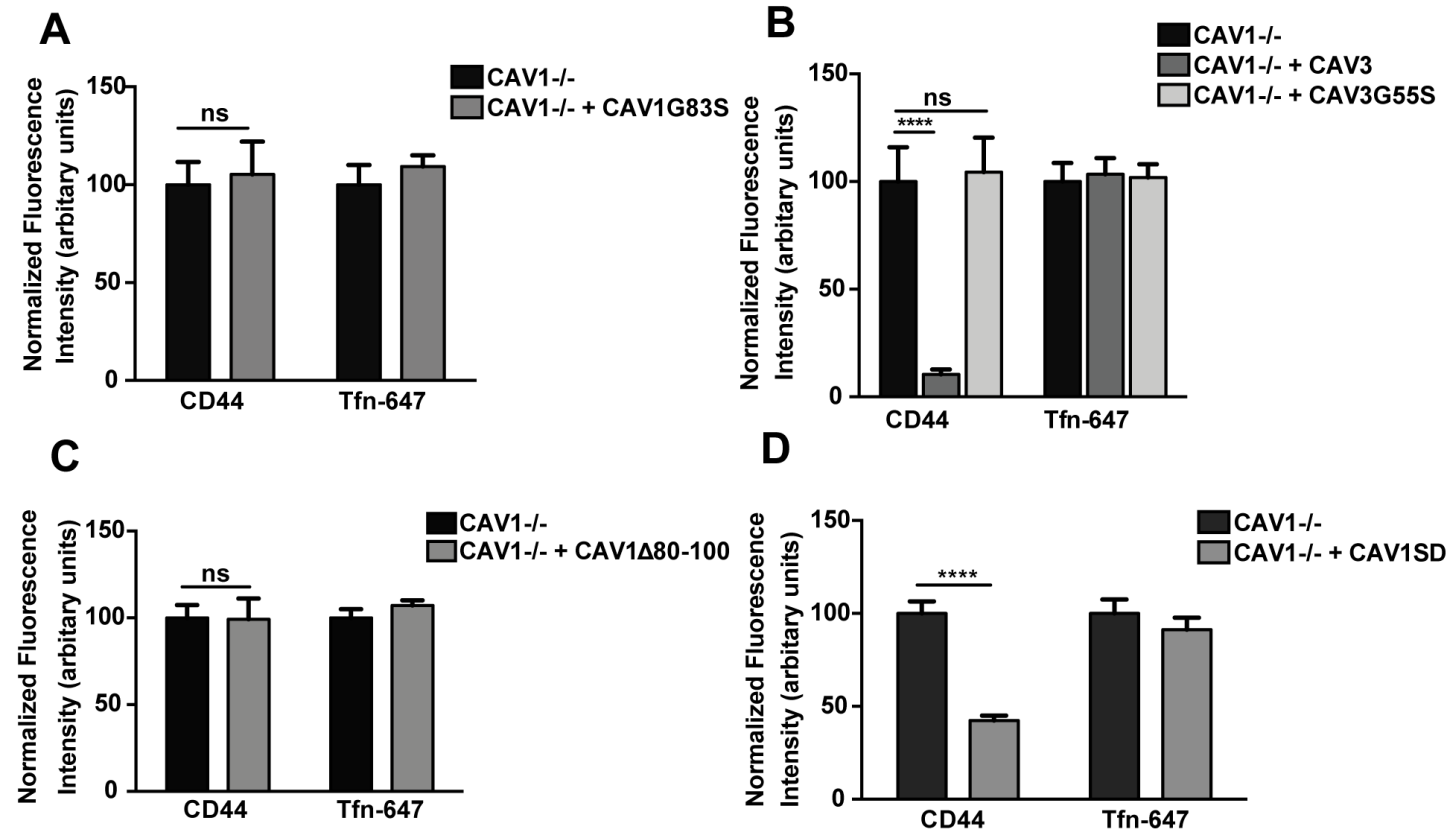
Scaffolding domain of CAV1 is crucial for inhibition of CLIC endocytosis. CAV1−/− MEFs were transiently transfected with (A) CAV1G83S-mCherry, (B) RFP-CAV3 and Cav3G55S-HA (C) CAV1Δ80-100-HA and (D) CAV1-SD-GFP (GFP-tagged minimal caveolin scaffolding domain) respectively. (A–D) Internalization assay was performed with anti-CD44 mAb and Tfn-647 for 2 min at 37°C. Cells were acid washed prior to fixation and internalized anti-CD44 mAb was labeled with AF-555 secondary antibody. 40–50 cells from each transfection were quantified for normalized fluorescence intensity of endocytic markers. In (A–D) data represent mean ± SEM of three independent experiments. **p = 0.002,***p = 0.0007, ****p<0.0001 (two-tailed t-test).

To characterize the regulatory mechanism by which CAV1 inhibits the CLIC/GEEC pathway, we investigated the role of caveolin scaffolding domain (CSD) of CAV1, as this domain has been shown to mediate several regulatory roles of CAV1 [47],[48]. We tested CAV1 and CAV3 CSD point substitution mutants (CAV1G83S; CAV3G55S) and the deletion mutant (CAV1Δ80–100) as used in previous studies [43]. Expression of the CSD mutants, unlike the WT proteins, did not inhibit CD44 mAb internalization in CAV1−/− MEFs (Figure 5A–C), suggesting that the CSD is required for inhibition of the CLIC/GEEC pathway. We next investigated whether the CSD is sufficient for inhibition by expressing the minimal CAV1 scaffolding domain as a fusion protein with GFP (CAV1-SD, amino acid 82–101) in CAV1−/− MEFs. CD44 mAb uptake was significantly decreased (58±3% decrease; mean ± SEM) in cells expressing CAV1-SD while Tfn-647 uptake was unaffected (Figure 5D). Taken together, these results suggest that inhibition of the CLIC/GEEC pathway by caveolin proteins requires an intact scaffolding domain and that this domain alone has significant inhibitory activity on CLIC endocytosis. The fact that the scaffolding domain has inhibitory activity when expressed in cells lacking endogenous CAV1 shows that the inhibitory activity of the mutant is not mediated through inhibition of interactions of proteins with endogenous CAV1 and caveolae but is an inherent property of this polypeptide. This suggests that CAV1 may affect fundamental membrane properties, as investigated in the following section.

### Caveolin-1 Decreases the Mobility of Lipid Raft Markers at the Plasma Membrane

CAV1 has been shown to interfere with the mobility of membrane microdomain-associated proteins, which further blocks the integrin-mediated internalization of bacteria [49]. We investigated whether similar effects could underlie the influence of expressed CAV1 and cavins on the CLIC/GEEC pathway.

**Figure 6:**
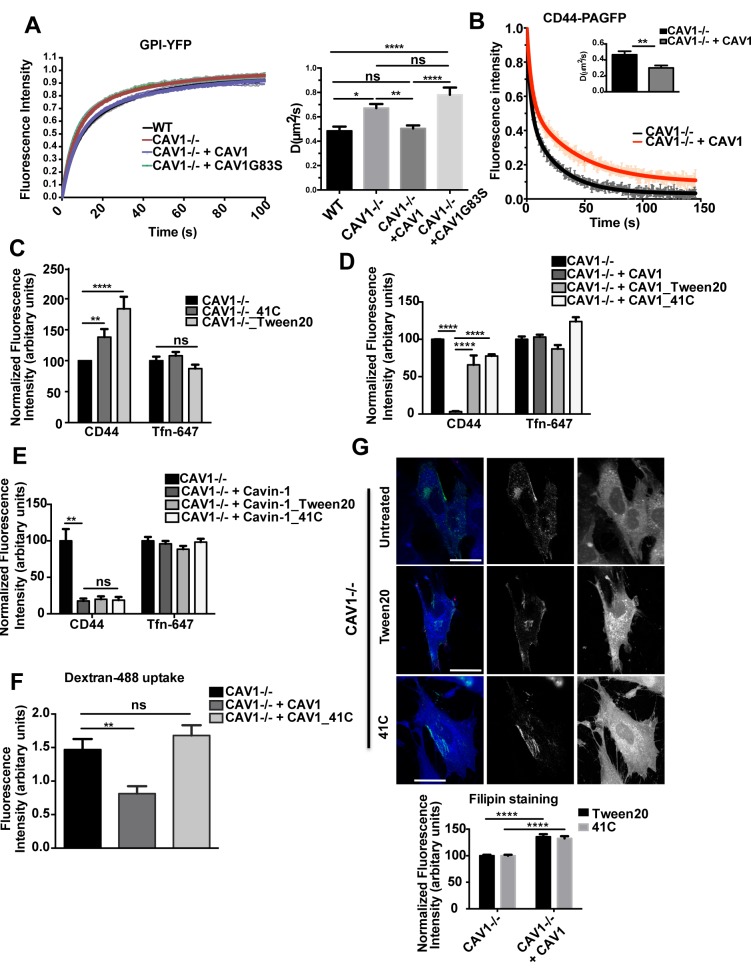
CAV1 alters general membrane characteristics. (A) WT and CAV1−/− MEFs were transiently transfected with GP1-YFP; CAV1−/− MEFs were also co-transfected with GPI-YFP plus CAV1-mCherry or CAV1G83S-mCherry. FRAP analysis for GPI-YFP diffusion was performed, and FRAP curves and diffusion coefficients (D) calculated from FRAP measurements in at least 30 cells per condition are shown. (B) CAV1−/− MEFs were transiently transfected with PA-CD44 and co-transfected with PA-CD44 and CAV1 respectively. Fluorescence decay curves and diffusion coefficients (D) for PA-CD44, calculated from photo-activation measurements in at least 20 cells per condition, are shown. (C) CAV1−/− MEFs were either treated with 0.05% Tween20 at 37°C prior to performing internalization assay with anti-CD44 mAb and Tfn-647 or incubated with endocytic markers at 41°C. Cells were acid washed prior to fixation and internalized anti-CD44 mAb was labeled with AF-555 secondary antibody. 40–50 cells from each treatment were quantified for normalized fluorescence intensity of endocytic markers. (D,E) CAV1−/− MEFs transiently expressing CAV1-YFP and Cavin-1-GFP respectively, were either treated with Tween20 or incubated with endocytic markers at 41°C. Uptake and quantification of internalized markers was performed as mentioned in (C). (F) In CAV1−/− MEFs transiently expressing CAV1-YFP, internalization assay were performed with Dex-488 for 5 min either at 41°C or at 37°C. 40–50 cells from each condition were quantified for fluorescence intensity of the internalized Dex-488. (G) Representative images for filipin labeling performed for CAV1-YFP-expressing treated and untreated CAV1−/− MEFs and a bar graph representing quantification for normalized fluorescence intensity of filipin are shown. In (A) and (B) data represent average mean ± SEM of pooled data from three independent experiments. Diffusion coefficients were obtained by non-linear regression of recovery and fluorescence decay curves, as described in the Materials and Methods section. In (C–F) data represent mean ± SEM of three independent experiments. *p<0.01, **p = 0.007, ***p<0.005, ****p<0.0001 (two-tailed t-test). Scale bar: 10 µm.

We first analyzed, using FRAP, the degree of mobility of a lipid raft-associated CLIC/GEEC cargo protein, GPI-YFP, in the presence and absence of CAV1. WT and CAV1−/− MEFs were transiently transfected with GPI-YFP or co-transfected with GPI-YFP and CAV1-mCherry. To analyze the mobility of GPI-YFP, a small ROI was bleached at the PM, half-life times of fluorescence recovery were recorded and the resulting diffusion coefficients were calculated. When compared with WT MEFs or CAV1−/− MEFs expressing CAV1, a significant increase was observed in the mobility of GPI-YFP in CAV1−/− MEFs (diffusion coefficient for WT: 0.48±0.03; CAV1−/−+CAV1: 0.50±0.02; CAV1−/− 0.67±0.03; mean ± SEM, n>20 cells, p<0.001) (Figure 6A). We also analyzed the mobility of a model GPI-anchored protein in CAV1−/− MEFs co-expressing GPI-YFP and the CSD mutant CAV1G83S. As expected, the mutant failed to decrease the mobility of GPI-YFP (diffusion coefficient 0.78±0.06) (Figure 6A), suggesting that localization of CAV1 at the PM with an intact scaffolding domain is required to restrict the mobility of the tested microdomain-associated proteins. The very short half-life times (around 8.5 seconds) observed in the above analysis make it unlikely that the changes are due to a defect in trafficking of GPI-anchored proteins, as exocytosis and endocytosis occur at longer time scales.

We next tested the effect of CAV1 on the mobility of the CLIC-specific cargo protein CD44. To characterize CD44 membrane diffusion ability, we made use of an expression construct generated by fusing a photo-activatable variant of green fluorescent protein (mRFP-PAGFP) to CD44 (mRFP-PAGFP-CD44, termed PA-CD44). First, we tested the internalization route of the PA-CD44 construct. PA-CD44 was photo-activated at a specific region of the PM of PA-CD44-expressing COS-7 cells. Internalized PA-CD44 showed significant colocalization with Dex-647-labeled endocytic vesicles in the photo-activated region consistent with endocytosis via the CLIC pathway (Figure S7, Movie S2). For diffusion studies, PA-CD44 was photo-activated in a small ROI at the PM and decay of fluorescence over time was measured in CAV1−/− MEFs and CAV1−/− MEFs expressing CAV1, respectively. In the presence of CAV1, the CD44 diffusion rate was significantly decreased in comparison with CAV1−/− MEFs and was on a similar time scale to that observed in the FRAP experiments (Figure 6B).

As the effects of CAV1 on CD44 and GPI-YFP may be mediated through its impact on physical properties of the PM, we then investigated whether stimulation of membrane fluidity by chemical or physical means would have any effect on internalization of cargo proteins of the CLIC/GEEC pathway using two independent treatments [49],[50]. As a chemical stimulus, we treated CAV1−/− MEFs with 0.05% Tween20 for 15 min at 37°C. After treatment, cells were incubated with anti-CD44 mAb and Tfn-647 for 2 min at 37°C. Secondly, we used high temperature, which can also alter the membrane mobility of proteins. CAV1−/− MEFs were incubated with anti-CD44 mAb and Tfn-647 for 2 min at 41°C. Interestingly, both physical and chemical stimuli caused a significant increase in CD44 mAb uptake while Tfn-647 uptake was unaffected, compared with untreated cells (Figure 6C). We further analyzed whether alterations in membrane fluidity or mobility of microdomain-associated proteins could overcome the inhibitory effect of CAV1 and Cavin-1 on their internalization by the CLIC/GEEC pathway. CAV1−/− MEFs were transiently transfected with CAV1-YFP and Cavin-1-GFP respectively. Cells were either treated with Tween20 prior to uptake or incubated with endocytic markers at 41°C, as described above. Both treatments significantly increased CD44 mAb uptake, overcoming the inhibitory effect of CAV1 (Figure 6D). In contrast the treatments did not affect endocytosis in Cavin-1 expressing cells (Figure 6E). Tfn-647 internalization was also unaffected in both treated and untreated cells (Figure 6D,E). We also tested the effect of an increase in membrane fluidity on fluid phase endocytosis. CAV1−/− MEFs were transiently transfected with CAV1-mCherry, and Dex-488 uptake (5 min) was performed at 41°C. At 41°C, Dex-488 uptake was unaffected by CAV1 expression, suggesting that higher temperature rescues the inhibition of both a specific CLIC marker and a fluid phase marker (Figure 6F). Taken together, these results suggest that CAV1 expression can modulate the mobility of membrane microdomain-associated proteins. Treatments that increase the mobility of these proteins can at least partially restore endocytosis through the CLIC pathway.

The physical and chemical stimuli described above alter mobility or fluidity of microdomain-associated proteins, an effect that might indicate lipid changes in the PM. As cholesterol is crucial for the function of the CLIC pathway [30], we investigated the effect of these treatments on cellular cholesterol distribution using the polyene antibiotic, filipin. CAV1−/− MEFs were transiently transfected with CAV1-YFP, treated with Tween20 or incubated with endocytic markers at 41°C, and labeled with filipin. In CAV1-expressing and treated cells, higher filipin labeling was observed (135.7±4.8% for Tween20 and 132.7±4.1% for 41°C; mean ± SEM) in comparison with treated, untransfected CAV1−/− MEFs (100±1.6%; mean ± SEM) (Figure 6G) or untreated CAV1-expressing cells. This suggests that CAV1 expression affects membrane cholesterol distribution/availability, which inhibits internalization via the CLIC/GEEC pathway. The physical and chemical stimuli restore cholesterol distribution allowing up-regulation of the CLIC/GEEC pathway. This effect is more complex than simply the availability of free cholesterol in the bulk membrane because cholesterol addition in the form of a cholesterol–cyclodextrin complex did not rescue the CAV1-mediated inhibition (unpublished data).

### Cavin-1 Influences Cholesterol Distribution, Cdc42 Activity, and Endosomal Sorting

**Figure 7:**
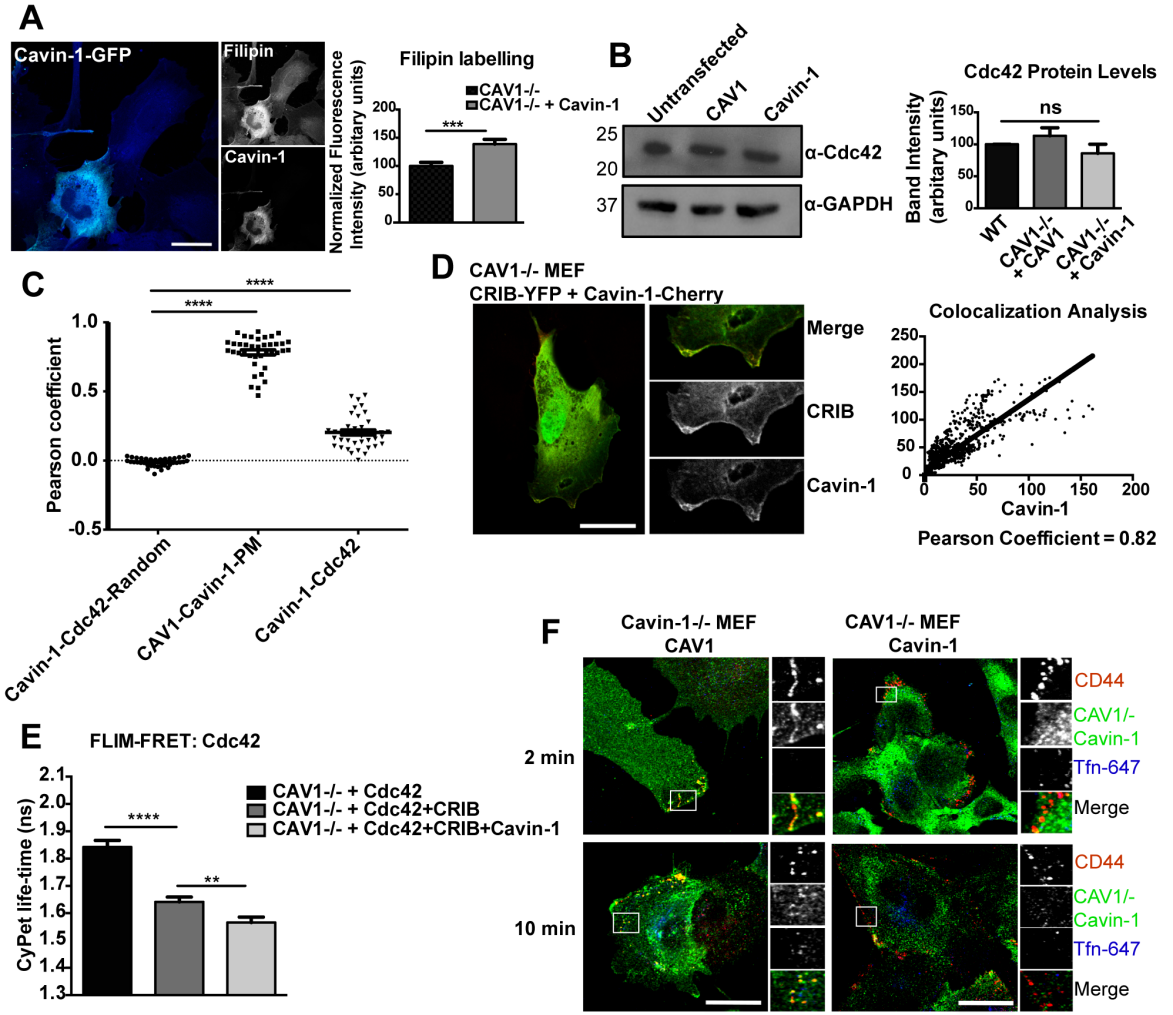
Cavin-1 regulates cholesterol distribution and Cdc42 activity. (A) Filipin labeling was performed for Cavin-1-GFP-expressing CAV1−/− MEFs, and for quantification average fluorescence intensity measured from 40–50 transfected cells was normalized to untransfected cells. (B) Whole cell lysates from CAV1−/− MEFs expressing CAV1-YFP and Cavin-1-GFP respectively were immunoblotted for Cdc42 and glyceraldehyde 3-phosphate dehydrogenase (GAPDH) was used as a loading control. The bar graph represents quantitation of protein levels, calculated by measuring band intensities by densitometry. (C) Pearson coefficient analysis determined at the plasma membrane for CAV1/Cavin-1 (positive control), Cavin-1/Cdc42, and randomized Cavin-1/Cdc42. (D) CAV1−/− MEFs were co-transfected with CRIB-YPet and Cavin-1-mCherry and quantitative colocalization analysis was performed at the PM ruffles. For 30 cells, line scans (10 pixels' length) were performed at random positions on PM ruffles and cytosol. The resultant fluorescent intensities profiles were plotted against each other and subjected to linear regression analysis. (E) CAV1−/− MEFs were transiently transfected with Cdc42-CyPet alone, with Cdc42-CyPet and CRIB-YPet simultaneously, or with Cdc42-CyPet, CRIB-YPet, and Cavin-1-Flag. Lifetime of CyPet was analyzed by FLIM-FRET and a representative graph showing lifetime of CyPet in 25 cells, mean ± SEM, is shown. (F) In Cavin-1−/− and CAV1−/− MEFs internalization assay was performed with anti-CD44 mAb and Tfn-647 for 2 and 10 min at 37°C. Endogenous CAV1 and Cavin-1 was labeled with respective primary antibodies followed by AF-488 secondary antibody labeling and for internalized anti-CD44 mAb labeling AF-555 secondary antibody was used. In (A,B,D) data represent mean ± SEM of three independent experiments. In (C) data represent average mean ± SEM of pooled data from three independent experiments; n = 40 cells. **p<0.01,***p<0.001 ****p<0.0001 (two-tailed t-test). Scale bar: 10 µm.

Unlike CAV1, physical and chemical stimuli could not rescue the internalization of CD44 mAb in Cavin-1-expressing cells (Figure 6E), suggesting Cavin-1 might not act by regulating the mobility of microdomain-associated surface proteins in these cells. Cavins have also been reported to respond to cholesterol levels and alter cholesterol cellular distribution [51],[52]. Thus, we next tested whether expression of Cavin-1 in cells lacking CAV1 could affect cellular cholesterol distribution by labeling Cavin-1-GFP-expressing CAV1−/− MEFs with filipin to analyze the distribution of free cholesterol. In Cavin-1-expressing cells we observed a significant increase (138±8.4%; mean ± SEM) in filipin labeling in comparison with untransfected cells (100±6.5%; mean ± SEM) (Figure 7A). These results suggest that Cavin-1 expression can alter the distribution of filipin staining, indicating a change in distribution of cholesterol in cells.

To examine the possible implications of such alteration in lipid distribution, we focused on Cdc42, whose localization and activation has been shown to depend on specific lipid organization at the PM [53]. CAV1−/− MEFs were transiently transfected with Cavin-1-GFP and endogenous Cdc42 protein levels were analyzed by Western blotting. No effect was observed on total Cdc42 protein levels in Cavin-1-expressing cells (Figure 7B, Figure S8). We next characterized the correlation between Cavin-1 and Cdc42 at the PM. CAV1−/− MEFs expressing Cavin-1-GFP were labeled for endogenous Cdc42 and subjected to Pearson coefficient analysis to calculate the correlation between the two proteins. This revealed a significant spatial correlation, compared with randomized values, between Cdc42 and Cavin-1 at the cell surface, although less than that between CAV1 and Cavin-1 (Cavin-1–Cdc42: 0.21±0.02, randomized: −0.01±0.01, CAV1–Cavin-1 PM: 0.78±0.02; mean ± SEM, n = 40, p<0.0001) (Figure 7C). This suggested that Cdc42 and Cavin-1 might colocalize within regions of the cell surface. Indeed, live-cell imaging of CAV1−/− MEFs co-transfected with Cdc42-GFP and Cavin-1-mCherry revealed that these two proteins co-accumulated in PM ruffles (Figure S9A). Further live-cell imaging demonstrated that GPI-YFP also co-accumulated with Cavin-1-mCherry in PM ruffles, suggesting that these are sites for the possible association of Cavin-1 with CLIC components (Figure S9B).

We next tested whether Cavin-1 also affected Cdc42 activity, using a fluorescently tagged CRIB domain (Cdc42/Rac-interacting binding domain from N-WASP). This construct (CRIB-YPet) binds to GTP-loaded Cdc42 and thus can act as a location biosensor for active Cdc42. CAV1−/− MEFs were either transfected with CRIB-YPet alone or co-transfected with both CRIB-Ypet and Cavin-1-mCherry. Quantitative line scan analysis of fluorescence intensity showed a high degree of colocalization between CRIB and Cavin-1 at the PM ruffles in comparison with cytosol (Pearson coefficient, PM: 0.82±0.04; cytosol: 0.35±0.01; mean ± SEM, p<0.001) (Figure 7D). This supported the notion that Cavin-1 colocalized with active Cdc42 in ruffles.

We then tested whether Cdc42 could be activated by Cavin-1 expression. We used the FRET pair of Cdc42-CyPet and CRIB-YPet, whose association on GTP-loading of Cdc42 leads to a decrease in the lifetime of CyPet that can be measured by FLIM-FRET. CAV1−/− MEFs were co-transfected with Cdc42-CyPet and CRIB-YPet either in the presence or absence of Cavin-1. The lifetime of CyPet was significantly reduced, consistent with energy transfer in Cavin-1-expressing cells (1.5±0.02 nanoseconds), compared with cells lacking Cavin-1 (1.64±0.01 nanoseconds; mean ± SEM) (Figure 7E). Therefore Cavin-1 expression promotes activation of Cdc42 in CAV1-deficient cells. All together these results suggest that Cdc42 is selectively activated in PM ruffles when non-caveolar Cavin-1 is expressed in cells.

We also noted additional cellular effects upon the loss of Cavin-1. In WT MEFs, neither CAV1 nor Cavin-1 colocalized significantly with either anti-CD44 mAb or Tfn-647 after 2 or 10 min of uptake (Figure 1A). However, in Cavin-1−/− MEFs colocalization was observed between CAV1 and the internalized CD44 mAb but not with Tfn-647 after 2 min of uptake (Figure 7F). We further confirmed this association of CAV1 with CLICs by assessing its localization following inhibition of either CLICs or CME. Upon inhibition of CME by the small molecule dynamin inhibitor dyngo4a, CAV1 still colocalized with the CD44 mAb, whereas this colocalization was lost when the CLIC/GEEC pathway was inhibited by 7-KC (Figure S10). This suggests that noncaveolar caveolin is preferentially recruited into the CLIC/GEEC pathway in cells lacking Cavin-1.

Loss of Cavin-1 also affects trafficking through the CLIC/GEEC pathway. After 10 min of uptake in the Cavin-1−/− MEFs, internalized CD44 mAb and Tfn-647 were seen in the same endocytic vesicles (Figure 7F); this was not observed in WT or in CAV1−/− MEFs. These results provide evidence for a novel role for caveolar proteins in regulating not only the magnitude but also specific features of the endosomal system. In this respect we noted a significant increase in the expression levels of Tfn receptor in Cavin-1−/− MEFs (Figure S11).

### Physiological Regulation of CLIC Endocytosis by Caveolins and Cavins

**Figure 8:**
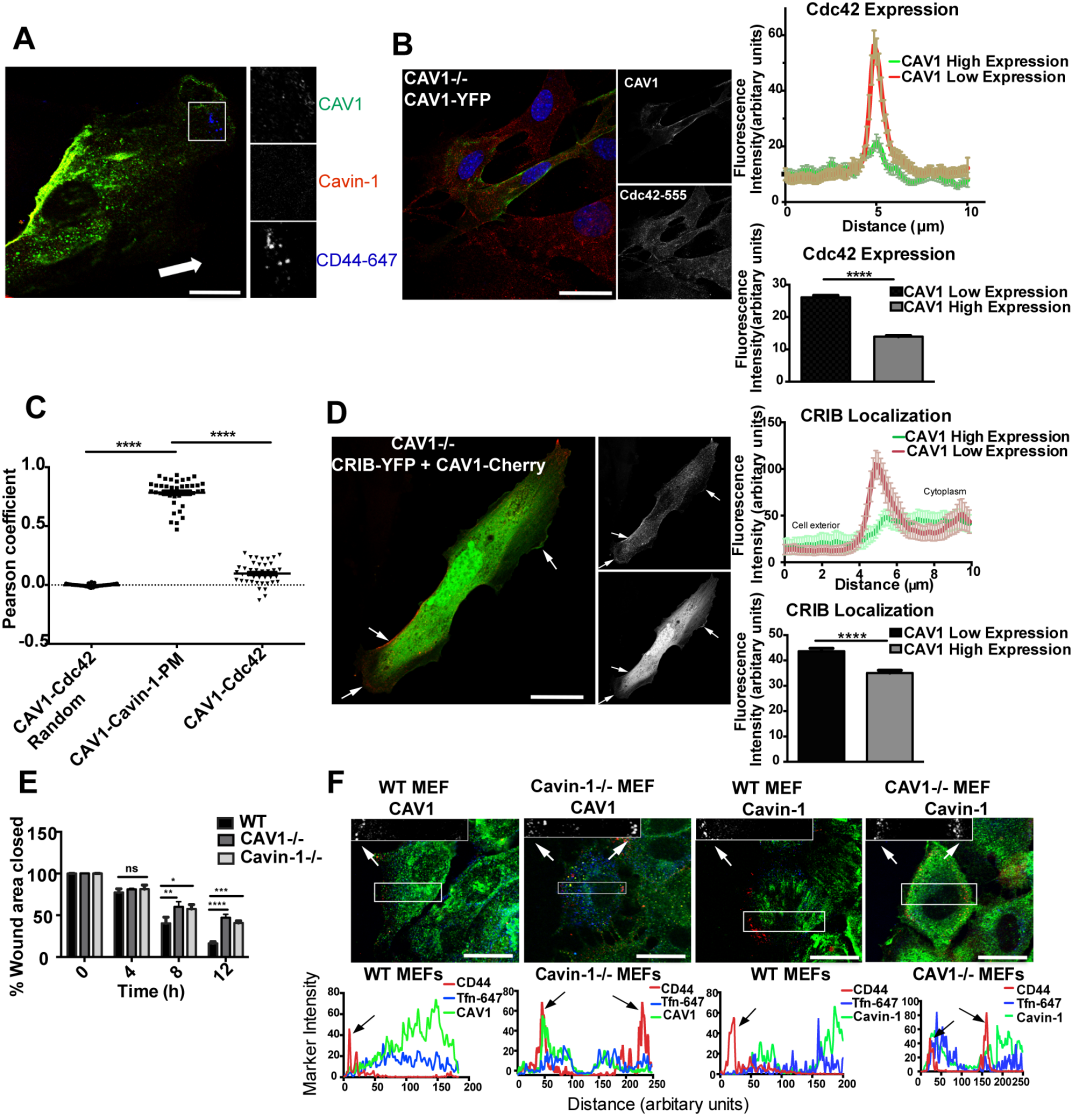
Physiological consequences of caveolin/cavin loss. (A) Confluent monolayer of CAV1−/− MEFs co-expressing CAV1-YFP and Cavin-1-mCherry was subjected to a scratch-wound assay. Internalization assay was performed with anti-CD44 mAb for 2 min at 37°C. Cells were acid washed before fixation and internalized anti-CD44 mAb was labeled with AF-647 secondary antibody. Arrow indicates direction of wound. (B) CAV1−/− MEFs expressing CAV1-YFP were labeled for Cdc42 with specific primary antibody followed by AF-555 secondary antibody labeling. For quantitative analysis of Cdc42 expression, line scans were performed in 30–40 CAV1-YFP-expressing cells at random positions on the PM. The plot profile represents average fluorescence (± SEM) of Cdc42 labeling in mentioned areas of PM from all cells and the bar graph represents amplitude together with error obtained from nonlinear Gaussian analysis. (C) Pearson coefficient analysis of the image data set from (B). (D) CAV1−/− MEFs were co-transfected with CRIB-Ypet and CAV1-mCherry and quantitative colocalization analysis was performed at the random regions of PM. For 20–30 co-transfected cells, line scans and nonlinear Gaussian analysis were performed as mentioned in (B) at random positions on the PM. (E) WT, CAV1−/−, and Cavin-1−/− MEF monolayers were subjected to a scratch-wound assay and the extent of migration of respective cells into the wound area was measured at 0, 4, 8, and 12 h time points. Percentage of wound closure is plotted. (F) At time points 0, 4, 8, and 12 h a scratch-wound assay, followed by internalization assay, was applied to WT, CAV1−/−, and Cavin-1−/− MEFs as mentioned in (A). For labeling endogenous CAV1 and Cavin-1 respective primary antibodies followed by AF-488 secondary antibody were used. The plot profile represents average pixel intensity across the leading to trailing edge for CD44, CAV1, and Tfn-647. Arrows indicate CD44 labeling. In (C) data represents mean ± SEM of data pooled from three independent experiments; n = 40 cells. In (B, D, E) data represent mean ± SEM of three independent experiments. *p<0.05, **p<0.01, ***p = 0.001, ****p<0.0001 (two-tailed t-test). Scale bar: 10 µm.

The inhibitory roles of caveolae, caveolins, and cavins suggest an important role for caveolae in regulating the CLIC/GEEC pathway. We therefore examined the physiological consequences of this inhibition. We first examined whether caveolae could play a role in spatial organization of the CLIC/GEEC pathway in migrating cells. A scratch-wound assay was applied to a confluent monolayer of CAV1−/− MEFs co-expressing CAV1 and Cavin-1. Fluorescently tagged CAV1 and Cavin-1 showed complete colocalization at the rear of the migrating cells (Figure 8A), consistent with previous studies showing caveolae enriched in this domain [38],[54]. CLIC endocytosis as detected by CD44 mAb uptake was dramatically reduced by the co-expression of the two caveolar proteins but the few carriers observed were invariably in areas lacking caveolae (Figure 8A). Concomitantly, in confluent monolayers of CAV1-expressing CAV1−/− MEFs, we also checked the cellular distribution of Cdc42, a pivotal regulator of polarity and the CLIC/GEEC pathway. In CAV1-YFP-expressing cells Cdc42 expression, detected by a Cdc42-specific antibody, was observed to be excluded from CAV1-positive areas. Quantification of fluorescence intensity at the cell surface, by line scan analysis, showed a significant decrease in Cdc42 expression in regions of PM expressing high levels of CAV1 compared with low- or nonCAV1-expressing regions (CAV1 high expression region: 14±0.32; low/no expression regions: 26±0.60; mean ± SEM, p<0.0001), within the same cell (Figure 8B). However, overall Cdc42 protein levels were unaltered in CAV1-YFP-expressing cells, as observed by Western blotting (Figure 7B, Figure S8).

To further characterize the correlation between Cdc42 and CAV1 at the cell surface, we performed the Pearson correlation coefficient analysis on the same set of images that were used for line scan analysis. This revealed significantly less correlation between CAV1 and Cdc42 at the PM in comparison with CAV1 and Cavin-1 at the PM (Pearson coefficient, CAV1–Cdc42: 0.11±0.01, randomized: −0.002±0.001, CAV1–Cavin-1 PM: 0.78±0.02; mean ± SEM, n = 40, p<0.0001) (Figure 8C). As expected from the above results, the activity of Cdc42, monitored by recruitment of the CRIB domain, was also negatively affected by CAV1 expression. Quantitative line scan analysis showed significantly fewer CRIB protein in regions of the PM expressing high levels of CAV1 in comparison with lower-expressing regions (PM region enriched in CAV1: 45±1.9; PM regions with low/no CAV1 expression: 57±2.4; mean ± SEM, p<0.001) within the same cell (Figure 8D). This suggests that CAV1 expression can result in differential distribution of CLIC components; in addition, caveolae can locally inhibit CLIC endocytosis and help polarize the pathway to the leading edge.

To test this hypothesis, we next examined the effect of loss of caveolar proteins on polarization of CLICs to the leading edge of 2D migrating fibroblasts, a key feature of the CLIC/GEEC pathway [29]. First we analyzed whether lack of caveolar proteins can alter cell migration by using a scratch-wound assay applied to confluent monolayers of WT, CAV1−/−, and Cavin-1−/− MEFs. As shown in a previous study [55], we observed a significant decrease in ability of CAV1−/− MEFs to close the wound compared with WT MEFs (after wound closure at 12 h, WT: 84±2.1%; CAV1−/−: 54±4.1%; mean ± SEM, p<0.0001) (Figure 8E). Similarly, Cavin-1−/− MEFs also showed less efficient wound closure compared with WT MEFs and showed defects in cell migration (after wound closure at 12 h, Cavin-1−/−: 60±2.5%; mean ± SEM, p<0.001) (Figure 8E). To characterize the polarization of CLICs in migrating cells, confluent monolayers of WT, CAV1−/−, and Cavin-1−/− MEFs were scratch-wounded and uptake of CD44 mAb and Tfn-647 was compared with the localization of CAV1 or Cavin-1 as cells migrated into wound. In both CAV1−/− and Cavin-1−/− MEFs, CD44 mAb internalization occurred at both the leading and trailing edge, indicating the CLIC/GEEC endocytosis was no longer polarized in these cells (Figure 8F).

**Figure 9:**
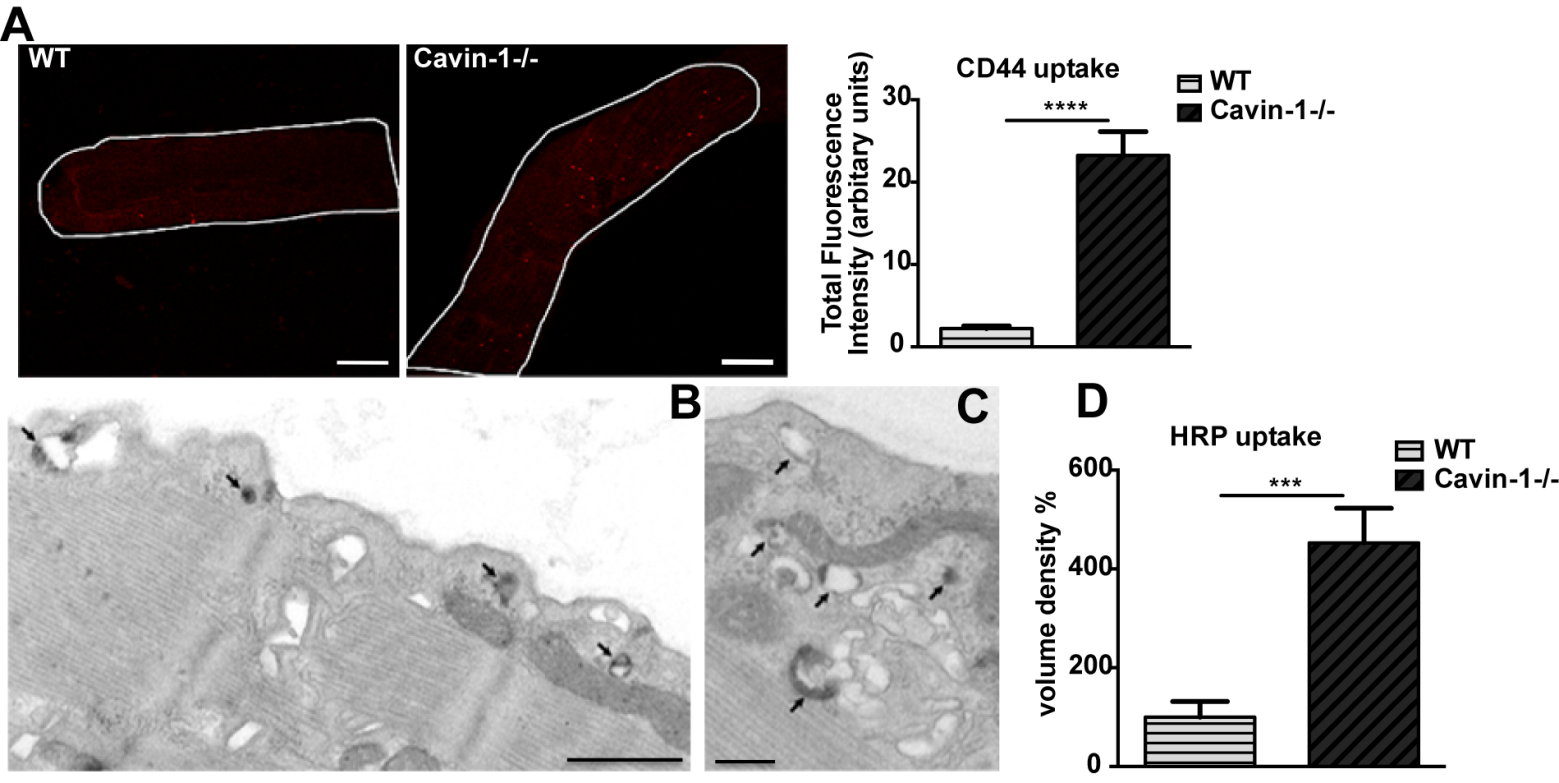
Loss of Cavin-1 up-regulates endocytic activity in muscle fibers. (A) Muscle fibers, isolated from Cavin-1−/− and WT mice, were incubated with anti-CD44 mAb for 3 min at 37°C. Fibers were acid washed on ice and internalized anti-CD44 mAb was labeled with AF-555 secondary antibody. 8–10 muscle fibers from three independent experiments were quantified for total fluorescence intensity of internalized CD44 mAb. (B,C) HRP uptake was performed in WT (B) and Cavin-1−/− (C) muscle fibers followed by DAB reaction. Arrows indicate DAB-positive intracellular structures. (D) Quantitation of DAB-labeled structures, as described in the Materials and Methods section, is shown. In (A, D) data represent mean ± SEM of three independent experiments. ***p<0.001, ****p<0.0001 (two-tailed t-test). Scale bars: (A) 10 µm, (B) 1 µm, (C) 500 nm.

Loss of caveolins and cavins has profound effects on specific tissues in vivo, including skeletal and striated muscle (reviewed by [56]). To examine whether the loss of caveolar components also affected endocytic activity in a differentiated tissue relevant to disease, we isolated mature adult muscle fibers from the flexor digitorum brevis muscle of WT and Cavin-1−/− mice. We assessed endocytic activity by adding either anti-CD44 mAb to the isolated fibers and visualizing uptake by IF, or by using HRP as a fluid phase marker for EM analysis. No significant difference was seen in CD44 surface labeling of the fibers, suggesting endogenous levels and surface accessibility of CD44 are not altered (not shown). CD44 mAb internalization was dramatically increased in fibers isolated from Cavin-1−/− mice (23.4±2.9, compared with WT: 2.2±0.3; mean ± SEM) (Figure 9A). Similarly, isolated fibers incubated with HRP as a fluid phase marker showed a highly significant 4.5-fold increase in the volume of HRP-labeled structures, as compared with WT fibers (Figure 9B–D), indicative of greatly increased fluid phase endocytosis. Taken together, these data show that the CLIC/GEEC pathway is fundamentally altered by the loss of Cavin-1 demonstrating a dramatic in vivo consequence of loss of caveolar components.

## Discussion

In this study we have identified extensive crosstalk, at several levels, between the caveolae and the CLIC/GEEC endocytic pathway in mammalian cells. Previous studies have shown that caveolin over-expression inhibits CIE [23],[39]. We now show that both expression comparable with physiological levels and down-regulation of caveolin can regulate endocytosis, demonstrating that CAV1 is an important cellular endocytic regulator. Previous studies suggested a role for Tyr14 phosphorylation of caveolin in inhibition of plasma membrane Cdc42 required for fluid phase endocytosis [39] but this cannot explain the findings presented here. Firstly, CAV3, which lacks a tyrosine residue equivalent to Tyr14, is an equally potent inhibitor of CIE as is CAV1. Secondly, we observed no significant CAV1 colocalization with Cdc42. Finally, expression of the isolated caveolin scaffolding domain as a fusion protein with GFP also significantly inhibited the CLIC/GEEC endocytosis. This inhibition was observed even in cells lacking endogenous caveolins, arguing against an effect on caveolin-signaling protein interactions. These results hinted at a more general mechanism of inhibition, and in view of previous studies [49],[57] prompted us to test whether specific properties of the plasma membrane were affected by CAV1 expression. Expression of CAV1 significantly decreased the membrane mobility of CD44 and GPI-YFP membrane microdomain-associated CLIC cargo proteins. Interestingly, effects of CAV1 on both diffusion properties and internalization of CLIC cargo proteins required an intact scaffolding domain of CAV1, as inhibitory effects were lost with single-point mutations in this region. A role for CAV1, dependent on the scaffolding domain, has been reported in regulation of dynamin-dependent, raft-mediated internalization of the CTxB as well as in endocytosis of bacterial engaged integrins [48],[49]. It is therefore tempting to speculate that perturbation of membrane properties by CAV1 expression underlies the effect on the CLIC/GEEC endocytosis. We could significantly rescue the inhibition of CLIC/GEEC endocytosis exerted by CAV1 on CD44 mAb and fluid phase marker uptake by using previously characterized chemical and physical stimuli, which enhanced the fluidity of the PM [49]. Under these conditions, we also observed increased cellular staining for free cholesterol, but only in CAV1-expressing cells. We conclude that complex changes in cholesterol trafficking/accessibility may accompany CAV1 expression, specifically perturb CLIC/GEEC endocytosis through effects on membrane lipid composition, and be rescued by experimental manipulation of membrane fluidity. Independent studies from our laboratory have shown that loss of CAV1 also has striking effects on nanoscale organization of the plasma membrane, including increased clustering of phosphatidylserine and farnesylated K-ras but decreased nanoclustering of dually palmitoylated H-ras [58]. Additionally, quantitative EM analysis pointed at a direct inhibitory effect of CAV1 on early carrier formation in the CLIC/GEEC pathway. However, we cannot rule out additional CAV1 effects on the mobility of microdomain-associated cargo proteins affecting access into the CLIC pathway.

The effect of the 20-amino acid scaffolding domain of CAV1 on CIE indicates that this domain of caveolin can have potent biological activity even in cells lacking endogenous caveolin. The activities of corresponding peptides added to cells in the form of a cell-penetrating fusion protein are well described as potent activators of endothelial nitric-oxide synthase (eNOS) through inhibition of caveolin–eNOS interactions and implicated in regulation of Rac1 signaling, a putative CLIC-interacting protein, by CAV1 [29],[59],[60]. However, these effects are observed only in cells with endogenous caveolin [47], unlike the effects described here. Nevertheless, the potent effects of the isolated scaffolding domain on endocytic trafficking described here should be taken into account when evaluating the effects of these peptides in cells and tissues. In addition, the role of CAV1 in many signaling pathways could potentially be explained by the inhibition of this major endocytic pathway in view of the importance of endocytic trafficking in many signaling events [61]. Again, consistent with this, both inhibition of signaling [60],[62],[63] and CLIC endocytosis (this study; [39]) show a similar dependence on the caveolin scaffolding domain.

To our surprise, our studies also revealed a novel noncaveolar regulatory role for cavin family proteins, specifically Cavin-1 and Cavin-3. While expression of non-caveolar Cavin-1 inhibited the formation of CLIC/GEEC carriers we could not detect any changes in the physical properties of the membrane upon Cavin-1 expression, although more subtle changes below our detection limits certainly cannot be excluded. Instead, single cell-based analysis of Cavin-1 expressing CAV1−/− MEFs consistently demonstrated an increased intracellular filipin labeling. Our recent studies showed that Cavin-1 expression in prostate cancer cells that express endogenous CAV1-modulated secretion pathways and cholesterol distribution, with decreased levels of cholesterol and impaired recruitment of actin to the detergent resistant membranes [51]. In view of the dependence of the CLIC/GEEC pathway on actin and cholesterol [30], including perturbation by 7-KC, as shown in the present study, it is likely that Cavin-1 regulates the CLIC/GEEC pathway through effects on cholesterol trafficking and/or distribution. However, it is intriguing that this inhibition can occur in the absence of CAV1 and caveolae. In this context it is noteworthy that detailed real-time observations of Cavin-1 localization in CAV1−/− cells revealed colocalization with active Cdc42 and GPI-AP in membrane ruffles. Availability of phosphatidylserine (PS) at the PM has been shown to be critical for membrane localization and activation of Cdc42 [53], and previous work from our laboratory has shown that Cavin-1 can bind to the PS at PM [18]. Hence it seems plausible that by interacting with CLIC components/regulators at the PM Cavin-1 regulates the activity of the pathway. In support of this hypothesis we observed that Cavin-1 could modulate Cdc42 activity and so perturb the Cdc42 activation–deactivation cycle. This would be limiting for the activity of the CLIC/GEEC pathway, which is dependent on a functional Cdc42 cycle with inhibition by an activated form of Cdc42 [30],[33].

Cavin-1 appears to be a highly specific regulator of the CLIC/GEEC pathway in comparison with CME and may be a useful tool to further characterize the CLIC/GEEC endocytic route. In wild-type cells, CD44 is rapidly sorted into a distinct, transferrin-negative recycling route [29] but this sorting ability of CLICs was reduced in Cavin-1−/− cells, suggesting a novel role for Cavin-1 in endosomal sorting possibly through indirect effects on cholesterol. Similar observations were not made in CAV1−/− cells even though these cells have reduced Cavin-1 levels. An intriguing possibility is that the low levels of noncaveolar CAV1 in CAV1−/−, which we show is internalized predominantly through the CLIC/GEEC pathway, has a modulatory effect on sorting in the endocytic pathway.

In our system Cavin-3 also showed a potent and specific inhibition of the CLIC/GEEC endocytosis when expressed alone, while knockdown led to a significant increase in both CLIC/GEEC and clathrin-dependent endocytosis. A systemic study of endocytosis, exploiting multi-parametric image analysis and multi-dimensional gene profiling, has also implicated Cavin-3 in endocytic trafficking of transferrin [64]. Together, these studies suggest a more general role for Cavin-3 in regulation of endocytic trafficking.

Co-expression of caveolar proteins in cells generated caveolae and negatively regulated the CLIC/GEEC pathway. Our results suggest that association of caveolins and cavins with caveolae does not perturb their inhibitory activity and hence identifies cavins, caveolins and caveolae as regulators of CI endocytosis. What might be the function of this complex regulatory network? Caveolae are polarized in migrating cells and highly concentrated at the rear of the cell [18],[29],[38],[54]. In contrast, CLIC/GEEC endocytosis predominantly occurs at the leading edge of migrating cells [29]. In line with the above studies, we observed that expression and activation of the CLIC regulator, Cdc42, was differentially distributed in areas of the PM significantly devoid of CAV1 expression. Upon expression of CAV1 and Cavin-1, CLIC/GEEC endocytosis is reduced but residual endocytosis occurs exclusively in regions devoid of caveolae, suggesting potent local inhibition of endocytosis. Conversely, loss of CAV1 and/or Cavin-1 causes a reduction in polarized migration and polarization of CLIC/GEEC endocytosis leading to increased endocytosis at the rear of the cell. This suggests that polarization of the CLIC/GEEC pathway is dependent directly or indirectly on the caveolar membrane system and that caveolae can “dampen” endocytic activity at the rear of the cell. This model is further strengthened by examination of explant tissues from mice lacking caveolae due to genetic deletion of Cavin-1. WT skeletal muscle fibers show a remarkable density of caveolae and relatively low endocytic activity. Loss of caveolae in the skeletal muscle of Cavin-1−/− mice causes a dramatic increase in endocytic activity as monitored by CD44 mAb uptake and by fluid phase uptake.

It is now clear that the consequences of expression of caveolins or cavins are highly complex and modulation of membrane traffic and lipids must be taken into account in understanding their actions. The evolution of two sets of protein components associated with caveolae as independent inhibitory agents, when free or incorporated into caveolae, and the profound cellular consequences of their loss emphasizes the importance of the caveolar system as a key regulator of endocytosis in mammalian cells.

## Materials and Methods

### Ethics Statement

All the animal experiments were conducted in accordance with the guidelines of the ethics committee at The University of Queensland.

### Antibodies and Reagents

Mouse anti-CD44 (clone 5035-41.1D, Novus Biologicals), rabbit anti-CAV1 (BD Biosciences), rabbit anti-Caveolin2 (Sigma Aldrich), rabbit anti-Cavin-1 and Cavin-4 antibody were raised as described previously [16], rabbit anti-Cavin-3 (ProteinTech group), mouse anti-Cavin-2 (Sigma Aldrich), rabbit anti-HA (Sigma Aldrich), mouse anti-Cdc42 (Becton Dickinson), mouse anti-GFP (Roche), mouse anti-transferrin receptor antibody (Zymed), Alexa Fluor conjugated dextran (Life Technologies), anti-rabbit and anti-mouse Alexa Fluor antibodies (Invitrogen), Alexa Fluor conjugated transferrin (Invitrogen), Alexa Fluor conjugated phalloidin (Invitrogen), Filipin III (Sigma Aldrich), Dynasore (Sigma Aldrich), 7-Ketocholesterol (Sigma Aldrich), Protease Inhibitor Cocktail Set III (Merck Millipore), PhosSTOP Phosphatase Inhibitor Cocktail (Roche), Dyngo-4a (Sigma). Stealth RNAi siRNA duplex oligonucleotides targeted against mouse Cavin-1 (5′CCGCUGUCUACAAGGUGCCGCCUUU3′;5′AAAGGCGGCACCUUGUAGACAGCGG3′) and Cavin-3 (5′CCGGAGCUCUGAAGGCCCAUCAGAA3′; 5′UUCUGAUGGGCCUUCAGAGCUCCGG3′) (Invitrogen).

### Cell Culture

WT, immortalized CAV1-null (CAV1−/−), Cavin-1 null (Cavin-1−/−) mouse embryonic fibroblasts (MEFs), 3T3-L1 and COS-7 cells were maintained in Dubelcco's modified Eagle's medium (DMEM; Gibco) supplemented with 10% fetal bovine serum (FBS; Cambrix) and 2 mM L-Glutamine at 37°C and 5% CO_2_. For transfection, cells were either electroporated (BIORAD) or Lipofectamine 2000 (Invitrogen) was used, as per the manufacturer's instructions, and by this method 30–40% transfection efficiency was achieved. For siRNA transfection, Cavin-1 and Cavin-3-specific oligonucleotides were transfected at a final concentration of 600 pM using Lipofectamine 2000.

### Internalization Assay, Cell Migration, and Immunocytochemistry

Cells grown on 12 mm coverslips were placed on 50 µl pre-warmed drops containing 10 µg/ml anti-CD44 antibody/3 mg/ml Dex-488/10 µg/ml anti-GFP antibody+10 µg/ml Tfn-647 diluted in culture media and incubated at 37°C in a 5% CO_2_ incubator for time indicated. To remove any surface bound markers (CD44 mAb, GFP mAb, or Tfn-647), 2×30-second acid stripping was performed with 0.5 M glycine (pH 2.2), before fixation with 4% paraformaldehyde. The cell migration (scratch-wound) assay and immunocytochemistry were performed as previously described [29]. For cholesterol staining, cells were labeled with filipin, 50 µg/ml, for 2 h in the dark. For Cdc42 staining, cells were fixed with 10% TCA and quenched using 3×5-min washes of 30 mM glycine on ice. Permeabilized cells were blocked with 3% bovine serum albumin (BSA) and incubated in primary antibody overnight at 4°C followed by Alexa Fluor conjugated secondary antibody incubation for 40 min at room temperature.

### Electron Microscopy and Quantification of CLICs Carrier

In transfected CAV1−/− MEFs, HRP uptake (10 µg/ml) was performed at 37°C for 2 min, and after brief washing with DMEM containing 1% BSA, diaminobenzidine (DAB) (10 mg/ml) reaction was performed on the live cell as previously described in [29]. Fixation, embedding, and sectioning were performed as follows. MEFs were fixed with 2.5% glutaraldehyde in 0.1 M cacodylate buffer (pH 7.4). Cells were post-fixed with 1% osmium tetroxide for 1 h at room temperature and serially dehydrated with ethanol. Cells were embedded in increasing ratios of LX-112 resin∶ethanol to 100% resin, and polymerized overnight at 60°C. Ultrathin (60 nm) sections were cut on a Leica UC6 microtome and imaged on a JEOL1011 electron microscope at 80 kV. Quantifications were performed as follows: the perimeters of approximately 16 cells (per experimental condition) were imaged and the number of CLICs/GEEC carriers from each cell was quantified and averaged across all 16 cells. The average number of CLICs/GEEC carriers per cell was generated from two separate repeats of the same experimental conditions.

### Image Acquisition (for General and Live-Cell Imaging)

Cells were grown either on 12 mm coverslips or on 35 mm glass bottom dishes (Mat-Tek Corporation) and fluorescence micrographs were captured for random fields containing transfected cells on a confocal laser-scanning microscope (Zeiss 710 META; 510 META; Carl Zeiss Inc.). Images were captured with a 63× plan Apochromat 1.4 NA Oil objective (Zeiss, Jena, Germany), using a 488 nm laser line for excitation and a 505–530 nm band pass emission filter to capture GFP and Alexa Fluor 488 fluorescence, a 561 nm laser line for excitation, and a 580–620 nm band pass emission filter to capture RFP/mCherry/Alexa Fluor 555 fluorescence, and a 633 nm laser line for excitation and a 650 long pass emission filter for emission to capture Alexa Fluor 647 fluorescence. For live cell co-imaging of Cherry and YFP, YFP fluorescence was captured using the 488 nm laser line for excitation and a 505–530 nm band pass for emission. For live cell imaging a region of interest was chosen and 2× digital zoom was applied. Optical path and emission filters were applied as appropriate for each fluorophore. Images were processed with Adobe Photoshop CS3 (Adobe, San Jose, CA) and fluorescent intensity of fluorophores on images was measured using ImageJ (National Institutes of Health, Bethesda, MD). Where indicated, correlation between fluorescence intensities between two fluorophores in different subcellular location was determined using Pearson coefficient analysis. For this, binary masks (values 0 and 1) were created to isolate pixels belonging to different regions of interest (such as the plasma membrane and/or cytosol) by multiplying (pixel by pixel) the mask image by the raw images. This method was chosen in preference to the Pearson analysis over the entire cell as it provides a better estimation of whether or not a high correlation exists between different fluorophores at a specific subcellular location, for example the plasma membrane. Then, each individual image (two channels) was used to calculate the Pearson correlation coefficient between the two fluorophores in each specific subcellular location, and the data represent an average Pearson correlation coefficient determined for at least 40 cells from at least three independent experiments.

### FRAP Experiments

To assess GPI-YFP dynamics, cells were seeded on 35 mm glass bottom dishes and transfected with GPI-YFP construct [65]. Images were captured on an inverted confocal microscope using a 63× plan Apo 1.4 NA, Oil objective with 4× digital zoom, with a resolution of 0.15 µm/pixel. A circular region of interest (ROI, 4.7 µm radius) was bleached to ∼70% using the 488 nm argon laser line and 405 nm laser line at 100% transmission. Time-lapse images of the same region and a reference region of identical size were acquired before (20 frames, 5 seconds) and after (300 frames, 90 seconds) photobleaching with an interval of ∼250 milliseconds per frame using the 488 nm laser line at 2% transmission and emission detected between 500 and 530 nm. Half-life time and diffusion rate were calculated as described previously [49],[66]; also see Text S1 for details.

CAV1 dynamics were determined in Cavin-1−/− cells expressing CAV1-YFP alone or co-transfected with CAV1-YFP and Cavin-1-mCherry. Images were acquired as described above and half-times were calculated as described previously for CAV1-GFP in HeLa cells [67],[68]. The lateral diffusion nature of CAV1-YFP recovery curves at the plasma membrane was determined by performing FRAP experiments at variable ROI areas (9.6, 14.4, and 16.6 µm^2^) to analyze the dependency of half times with area size. Linear regression was performed and the diffusion coefficient for simple lateral diffusion was extracted from the slope as described in Text S1.

### Photoactivation Experiments

To achieve spatially defined photoactivation of PAGFP, a Ti:sapphire two-photon laser (1600–1800 mW Chameleon Ultra, Coherent Scientific) tuned at 775 nm was used [69]. For lateral diffusion studies of CD44, the plasma membrane region of cells was identified with the mRFP signal of CD44-mRFP-PA-GFP. PAGFP fluorescence was activated using a constant circular region of interest (ROI, 1.98 µm radius) by a single scan with infrared laser irradiation (30% transmission), and time-lapse images of photo-activated GFP at the same region were captured every ∼250 millisecond using a 60× plan Apo 1.4 NA, Oil objective at 4× digital zoom and appropriate filter sets to capture GFP fluorescence. For each time point, the average fluorescence intensity was calculated and normalized to the value at the first frame after photo-activation. Decay curves were fitted to a double exponential decay curve and a global half-time of fluorescence decay was obtained numerically as described for FRAP experiments.

### FLIM-FRET

CAV1−/− MEFs expressing FRET pair (Cdc42-CyPet and CRIB-YPet) either in presence or absence of FLAG-tagged Cavin-1 were subjected to FLIM microscopy as described previously [18].

### Dynamin Inhibition and 7-Ketocholesterol Treatment

Cells were serum starved for 3 h in serum-free DMEM before incubation with 60 µM dynasore/Dyngo-4a. Internalization assay was performed in the presence of 40 µM dynasore for the desired amount of time. Cells were treated with 30 µM 7-ketocholesterol (7-KC) for 30 min at 37°C, followed by the internalization assay.

### Western Blotting

Whole cell lysates were subjected to SDS-PAGE and further to Western blotting. Membranes were probed with primary antibody at the desired concentration for 1 h at room temperature or overnight at 4°C, followed by incubation with either secondary HRP-conjugated antibodies or infrared dye-labeled Odyssey secondary antibodies for 1 h at room temperature. For detection, either the Licor Odyssey infrared imaging system was used, as per the manufacturer's instructions (Licor Biotechnology), or the SuperSignal West Pico chemiluminescent substrate (Pierce) was captured on film (Kodak). Densitometric analysis of protein bands was performed either by ImageJ or by using Licor Odyssey analysis software. Also see Text S1 for details.

### Muscle Fiber Isolation and Analysis of Endocytic Activity

Muscle fibers were isolated from WT and Cavin-1−/− adult mice using a method described previously [70], with modifications. For EM analysis of HRP uptake, isolated fibers cultured overnight on matrigel-coated plastic dishes were incubated in HRP (10 mg/ml) at 37°C for 5 min, washed briefly, then fixed in glutaraldehyde before DAB visualization of the HRP reaction product. Quantitation of the volume of HRP-labeled elements relative to the sampled cytoplasmic volume (volume density) was determined by point counting of peripheral areas of WT and Cavin-1−/− muscle fibers, as shown in previous studies [29]. For procedure details see Text S1.

### Statistical Analyses

Statistical analyses were conducted using Microsoft Excel and Prism (GraphPad). Error bars represent either standard error of the mean (SEM) or standard deviation (SD) for at least three independent experiments, as indicated in figure legends. Statistical significance was determined either by two-tailed Student's t-test or by one-way ANOVA, as indicated in the figure legends.

## Supporting Information

Text S1 **Supplementary experimental procedures**
(DOCX)

Figure S1 **CD44 mAb as specific cargo of the CLIC/GEEC pathway.** (A) Internalization assay was performed in WT MEFs with anti-GFP mAb and Tfn-647 for 2 min at 37°C. Cells were acid washed prior to fixation and for labeling internalized GFP mAb secondary AF-488 antibody was used. (B) COS-7 cells were transiently transfected with CD44-GFP and internalization assay was performed with anti-CD44 mAb and Tfn-647 for 2 min at 37°C. Cells were acid washed prior to fixation and internalized anti-CD44 mAb was labeled with AF-555 secondary antibody. Arrows denote CD44 labeled puncta. Scale bar: 10 µm.
(TIF)

Figure S2 **CD44-GFP labeled vesicles co-localize with internalized Dex-647.** COS-7 cells transfected with CD44-GFP were imaged live at 37°C in presence of Dex-647 (2 mg/ml). Time-lapse covers a period of 7 min and images from the selected frames of the movie (Movie S1) are shown. Scale bar: 10 µm.
(TIF)

Figure S3 **Protein levels of caveolar components in WT, CAV1−/−, and Cavin-1−/− MEFs.** (A) Whole cell lysates from WT, CAV1−/− and Cavin-1−/− MEFs were immunoblotted with CAV1 and Cavin-1 primary antibodies followed by secondary HRP-conjugated antibodies. Actin was used as a loading control. For quantitative analysis of protein levels, Densitometric analysis of band intensities was performed. (B) Whole cell lysates from CAV1−/− and CAV1−/− expressing Cavin-1-specific siRNA were immunoblotted with Cavin-1 primary antibody followed by secondary HRP-conjugated antibodies. GAPDH was used as a loading control. A representative immunoblot is shown. The same set of transfected cells growing on coverslips were subjected to internalization assays with anti-CD44 mAb and Tfn-647 for 2 min at 37°C. Cells were acid washed prior to fixation. Internalized CD44 mAb was detected with an AF-555-labeled secondary antibody. The bar graph represents the quantification of internalized markers. Data represent mean ± SEM of three independent experiments.
(TIF)

Figure S4 **Reconstitution of CAV1 and Cavin-1 in CAV1−/− MEFs.** Whole cell lysates were prepared from WT, CAV1−/−, Cavin-1−/−, and CAV1−/− MEFs transiently transfected with CAV1-GFP and Cavin-1-GFP respectively. Lysates were immunoblotted with CAV1 and Cavin-1 primary antibodies followed by secondary fluorescent (Odyssey) antibodies. Actin was used as a loading control, and for detection the Licor Odyssey infrared imaging system was used.
(TIF)

Figure S5 **Inhibition of Dex-488 uptake by CAV1 and Cavin-1 in CAV1−/− MEFs.** (A) CAV1−/− MEFs were transiently transfected with CAV1-YFP and (B) with Cavin-1-GFP respectively. Internalization assay was performed with Dex-488 for 5 min at 37°C. 40–50 cells from each transfection from (A, B) were quantified for normalized fluorescent intensity of internalized Dex-488. Untransfected CAV1−/− MEFs represent control. In (A,B) data represent mean ± SEM of three independent experiments. ****p<0.0001 (two-tailed t-test). Scale bar: 10 µm.
(TIF)

Figure S6 **Cavin-mediated inhibition of the CLIC/GEEC pathway.** (A) CAV1−/− MEFs were transiently transfected with pIRES-Cavin-1, pIRES-Cavin-2, pIRES-Cavin-3 and pIRES-Cavin-4 respectively. Whole cell lysates from above transfected CAV1−/− MEFs, untransfected WT MEFs, untransfected CAV1−/− MEFs, and muscle tissue were immunoblotted with respective cavin primary antibodies followed by secondary HRP-conjugated antibodies. Lysates from untransfected CAV1−/− MEFs and WT were used as a control for Cavin-1–3 endogenous expression levels, while muscle lysates were used specifically as control for Cavin-4 endogenous expression. GAPDH was used as loading control. A representative Western blot is shown. The bar graphs represent densitometric analysis results of respective cavin protein levels (mean ± SEM; from three independent experiments) normalized to the values obtained in WT lysates. (B) 3T3-L1 cells were transiently transfected with siRNA directed to Cavin-1 and Cavin-3 respectively. 48 h post transfection cells lysates were immunoblotted with respective Cavin-1 and Cavin-3 primary antibodies followed by secondary HRP-conjugated antibodies. A representative Western blot is shown and lanes for control, Cavin-1 and Cavin-3 are cropped sections of the same film. The bar graph represents quantitation of Cavin-1 and Cavin-3 protein levels normalized to control levels, measured by densitometry. Actin was used as a loading control.
(TIF)

Figure S7 **Photo-activated CD44 (PA-CD44) labeled endocytic carriers co-localize with internalized dextran.** COS-7 cells were transfected with PA-CD44 and a selected ROI at PM was photo-activated and imaged at 37°C in presence of Dex-647 (2 mg/ml). Time-lapse covers a period of 7 min and images from the selected frames of the movie (Movie S2) are shown. Scale bar: 10 µm.
(TIF)

Figure S8 **CAV1-YFP and Cavin-1-GFP expression in CAV1−/− MEFs.** CAV1−/− MEFs were transiently transfected with CAV1-YFP and Cavin-1-GFP, respectively. Whole cell lysates were immunoblotted with CAV1 and Cavin-1 primary antibodies followed by secondary HRP-conjugated antibodies. GAPDH expression was used as a loading control.
(TIF)

Figure S9 **Cavin-1 co-localize with Cdc42 and GPI-AP at PM ruffles.** CAV1−/− MEFs were co-transfected with (A) Cdc42-GFP and Cavin-1-mCherry and with (B) GPI-YFP and Cavin-1-mCherry respectively, and cells were imaged live at 37°C. Time-lapse covers a period of 18 min and images from the selected frames of the movie are shown. Scale bar: 10 µm.
(TIF)

Figure S10 **Noncaveolar CAV1 is internalized** ***via*** **the CLIC/GEEC pathway.** Cavin-1−/−MEFs were either left untreated or treated with 60 µM Dyngo4a and 30 µM 7-KC respectively for 30 min prior to performing internalization assay with CD44 mAb and Tfn-647 for 2 min at 37°C. Endogenous CAV1 was labeled with respective primary antibodies followed by AF-488 secondary antibody labeling, and for internalized anti-CD44 mAb labeling AF-555 secondary antibody was used. Scale bar: 10 µm.
(TIF)

Figure S11 **Transferrin receptor levels in CAV1−/− and Cavin-1−/− MEFs.** Whole cell lysates from WT, CAV1−/− and Cavin-1−/− MEFs were immunoblotted with primary anti-transferrin receptor antibody followed by secondary HRP-conjugated antibodies. GAPDH was used as a loading control.
(TIF)

Figure S12 **Anti-CD44 mAb and Tfn-647 internalization in CAV1−/− MEFs.** CAV1−/− MEFs were incubated with anti-CD44 mAb and Tfn-647 for 2 min at 37°C. Cells were the placed on ice and acid washed before fixation. Internalized anti-CD44 mAb was labeled with AF-555 secondary antibody. Scale bar 10 µm.
(TIF)

Figure S13 **Anti-CD44 mAb and Tfn-647 internalization in CAV1-YFP expressing CAV1−/− cells.** CAV1−/− MEFs were transiently transfected with Cavin-1-GFP. Post-transfection cells were incubated with anti-CD44 mAb and Tfn-647 for 2 min at 37°C. Cells were then placed on ice and acid washed before fixation. Internalized anti-CD44 mAb was labeled with AF-555 secondary antibody. Scale bar 10 µm.
(TIF)

Figure S14 **Anti-CD44 mAb and Tfn-647 internalization in Cavin-1-GFP expressing CAV1−/− cells.** CAV1−/− MEFs were transiently transfected with Cavin-1-GFP. Post-transfection cells were incubated with anti-CD44 mAb and Tfn-647 for 2 min at 37°C. Cells were then placed on ice and acid washed before fixation. Internalized anti-CD44 mAb was labeled with AF-555 secondary antibody. Scale bar: 10 µm.
(TIF)

Movie S1 **CD44-GFP gets internalized** ***via*** **the CLIC/GEEC pathway.** COS-7 cells transiently expressing CD44-GFP were imaged live in culture media containing 2 mg/ml of Dex-647. The movie corresponds to the time-lapse series shown in Figure S2. Images were captured every 2.3 sec for 7 min. Scale bar: 5 µm.
(AVI)

Movie S2 **Photo-activated CD44 (PA-CD44) gets internalized** ***via*** **the CLIC/GEEC pathway.** COS-7 cells transiently expressing PA-CD44 were imaged live in culture media containing 2 mg/ml of Dex-647. The movie corresponds to the time-lapse series shown in Figure S7. Images were captured every 3.9 sec for 7 min. Scale bar: 5 µm.
(AVI)

## Abbreviations

7-KC: 7 ketocholesterol
AF: Alexa Fluor
CAV1: caveolin-1
CAV3: caveolin-3
CIE: clathrin independent endocytosis
CLIC: clathrin-independent carriers
CME: clathrin mediated endocytosis
CRIB: Cdc42/Rac-interacting binding domain
CSD: caveolin scaffolding domain
CTxB: cholera toxin subunit B
DAB: diaminobenzidine
EM: electron microscopy
eNOS: endothelial nitric-oxide synthase
FRAP: fluorescence recovery after photobleaching
GEEC: GPI-AP enriched early endosomal compartment
GPI-AP: glycosylphosphatidylinositol-anchored protein
mAb: monoclonal antibody
HRP: horseradish peroxidase
IF: immunofluorescence
PM: plasma membrane
PS: phosphatidylserine
Tfn: transferrin
WT MEFs: wild type mouse embryonic fibroblasts

## References

[1] Doherty GJ, McMahon HT (2009) Mechanisms of endocytosis. Annu Rev Biochem 78: 857–902. 19317650 10.1146/annurev.biochem.78.081307.110540

[2] Donaldson JG, Porat-Shliom N, Cohen LA (2009) Clathrin-independent endocytosis: a unique platform for cell signaling and PM remodeling. Cell Signal 21: 1–6. 18647649 10.1016/j.cellsig.2008.06.020 PMC2754696

[3] Hansen CG, Nichols BJ (2009) Molecular mechanisms of clathrin-independent endocytosis. J Cell Sci 122: 1713–1721. 19461071 10.1242/jcs.033951 PMC2723140

[4] Howes MT, Mayor S, Parton RG (2010) Molecules, mechanisms, and cellular roles of clathrin-independent endocytosis. Curr Opin Cell Biol 22: 519–527. 20439156 10.1016/j.ceb.2010.04.001

[5] Mayor S, Pagano RE (2007) Pathways of clathrin-independent endocytosis. Nat Rev Mol Cell Biol 8: 603–612. 17609668 10.1038/nrm2216 PMC7617177

[6] Parton RG, Simons K (2007) The multiple faces of caveolae. Nat Rev Mol Cell Biol 8: 185–194. 17318224 10.1038/nrm2122

[7] Schmid SL (2010) Clathrin-mediated endocytosis: a universe of new questions. Mol Biol Cell 21: 3818–3819. 21079028 10.1091/mbc.E10-05-0386 PMC2982115

[8] Oh P, Borgstrom P, Witkiewicz H, Li Y, Borgstrom BJ, et al (2007) Live dynamic imaging of caveolae pumping targeted antibody rapidly and specifically across endothelium in the lung. Nat Biotechnol 25: 327–337. 17334358 10.1038/nbt1292 PMC1979160

[9] Richter T, Floetenmeyer M, Ferguson C, Galea J, Goh J, et al (2008) High-resolution 3D quantitative analysis of caveolar ultrastructure and caveola-cytoskeleton interactions. Traffic 9: 893–909. 18397183 10.1111/j.1600-0854.2008.00733.x

[10] Schnitzer JE (2001) Caveolae: from basic trafficking mechanisms to targeting transcytosis for tissue-specific drug and gene delivery in vivo. Adv Drug Deliv Rev 49: 265–280. 11551399 10.1016/s0169-409x(01)00141-7

[11] Rizzo V, Morton C, DePaola N, Schnitzer JE, Davies PF (2003) Recruitment of endothelial caveolae into mechanotransduction pathways by flow conditioning in vitro. Am J Physiol Heart Circ Physiol 285: H1720–1729. 12816751 10.1152/ajpheart.00344.2002

[12] Thorn H, Stenkula KG, Karlsson M, Ortegren U, Nystrom FH, et al (2003) Cell surface orifices of caveolae and localization of caveolin to the necks of caveolae in adipocytes. Mol Biol Cell 14: 3967–3976. 14517311 10.1091/mbc.E03-01-0050 PMC206992

[13] Boucrot E, Howes MT, Kirchhausen T, Parton RG (2011) Redistribution of caveolae during mitosis. J Cell Sci 124: 1965–1972. 21625007 10.1242/jcs.076570 PMC3104031

[14] Fra AM, Williamson E, Simons K, Parton RG (1994) Detergent-insoluble glycolipid microdomains in lymphocytes in the absence of caveolae. J Biol Chem 269: 30745–30748. 7982998

[15] Parton RG, Hanzal-Bayer M, Hancock JF (2006) Biogenesis of caveolae: a structural model for caveolin-induced domain formation. J Cell Sci 119: 787–796. 16495479 10.1242/jcs.02853

[16] Bastiani M, Liu L, Hill MM, Jedrychowski MP, Nixon SJ, et al (2009) MURC/Cavin-4 and cavin family members form tissue-specific caveolar complexes. J Cell Biol 185: 1259–1273. 19546242 10.1083/jcb.200903053 PMC2712963

[17] Hansen CG, Bright NA, Howard G, Nichols BJ (2009) SDPR induces membrane curvature and functions in the formation of caveolae. Nat Cell Biol 11: 807–814. 19525939 10.1038/ncb1887 PMC2712677

[18] Hill MM, Bastiani M, Luetterforst R, Kirkham M, Kirkham A, et al (2008) PTRF-Cavin, a conserved cytoplasmic protein required for caveola formation and function. Cell 132: 113–124. 18191225 10.1016/j.cell.2007.11.042 PMC2265257

[19] McMahon KA, Zajicek H, Li WP, Peyton MJ, Minna JD, et al (2009) SRBC/cavin-3 is a caveolin adapter protein that regulates caveolae function. EMBO J 28: 1001–1015. 19262564 10.1038/emboj.2009.46 PMC2683698

[20] Stoeber M, Stoeck IK, Hanni C, Bleck CK, Balistreri G, et al (2012) Oligomers of the ATPase EHD2 confine caveolae to the plasma membrane through association with actin. EMBO J 31: 2350–2364. 22505029 10.1038/emboj.2012.98 PMC3364743

[21] Hansen CG, Howard G, Nichols BJ (2011) Pacsin 2 is recruited to caveolae and functions in caveolar biogenesis. J Cell Sci 124: 2777–2785. 21807942 10.1242/jcs.084319

[22] Hayer A, Stoeber M, Ritz D, Engel S, Meyer HH, et al (2010) Caveolin-1 is ubiquitinated and targeted to intralumenal vesicles in endolysosomes for degradation. J Cell Biol 191: 615–629. 21041450 10.1083/jcb.201003086 PMC3003328

[23] Kirkham M, Fujita A, Chadda R, Nixon SJ, Kurzchalia TV, et al (2005) Ultrastructural identification of uncoated caveolin-independent early endocytic vehicles. J Cell Biol 168: 465–476. 15668297 10.1083/jcb.200407078 PMC2171740

[24] Pelkmans L, Zerial M (2005) Kinase-regulated quantal assemblies and kiss-and-run recycling of caveolae. Nature 436: 128–133. 16001074 10.1038/nature03866

[25] Cheng ZJ, Singh RD, Sharma DK, Holicky EL, Hanada K, et al (2006) Distinct mechanisms of clathrin-independent endocytosis have unique sphingolipid requirements. Mol Biol Cell 17: 3197–3210. 16672382 10.1091/mbc.E05-12-1101 PMC1552047

[26] Sabharanjak S, Sharma P, Parton RG, Mayor S (2002) GPI-anchored proteins are delivered to recycling endosomes via a distinct cdc42-regulated, clathrin-independent pinocytic pathway. Dev Cell 2: 411–423. 11970892 10.1016/s1534-5807(02)00145-4

[27] Lamaze C, Dujeancourt A, Baba T, Lo CG, Benmerah A, et al (2001) Interleukin 2 receptors and detergent-resistant membrane domains define a clathrin-independent endocytic pathway. Mol Cell 7: 661–671. 11463390 10.1016/s1097-2765(01)00212-x

[28] Naslavsky N, Weigert R, Donaldson JG (2004) Characterization of a nonclathrin endocytic pathway: membrane cargo and lipid requirements. Mol Biol Cell 15: 3542–3552. 15146059 10.1091/mbc.E04-02-0151 PMC491817

[29] Howes MT, Kirkham M, Riches J, Cortese K, Walser PJ, et al (2010) Clathrin-independent carriers form a high capacity endocytic sorting system at the leading edge of migrating cells. J Cell Biol 190: 675–691. 20713605 10.1083/jcb.201002119 PMC2928008

[30] Chadda R, Howes MT, Plowman SJ, Hancock JF, Parton RG, et al (2007) Cholesterol-sensitive Cdc42 activation regulates actin polymerization for endocytosis via the GEEC pathway. Traffic 8: 702–717. 17461795 10.1111/j.1600-0854.2007.00565.x PMC7617178

[31] Gauthier NC, Monzo P, Kaddai V, Doye A, Ricci V, et al (2005) Helicobacter pylori VacA cytotoxin: a probe for a clathrin-independent and Cdc42-dependent pinocytic pathway routed to late endosomes. Mol Biol Cell 16: 4852–4866. 16055501 10.1091/mbc.E05-05-0398 PMC1237088

[32] Kalia M, Kumari S, Chadda R, Hill MM, Parton RG, et al (2006) Arf6-independent GPI-anchored protein-enriched early endosomal compartments fuse with sorting endosomes via a Rab5/phosphatidylinositol-3′-kinase-dependent machinery. Mol Biol Cell 17: 3689–3704. 16760436 10.1091/mbc.E05-10-0980 PMC1525230

[33] Kumari S, Mayor S (2008) ARF1 is directly involved in dynamin-independent endocytosis. Nat Cell Biol 10: 30–41. 18084285 10.1038/ncb1666 PMC7617176

[34] Lundmark R, Doherty GJ, Howes MT, Cortese K, Vallis Y, et al (2008) The GTPase-activating protein GRAF1 regulates the CLIC/GEEC endocytic pathway. Curr Biol 18: 1802–1808. 19036340 10.1016/j.cub.2008.10.044 PMC2726289

[35] Fielding CJ, Fielding PE (2001) Caveolae and intracellular trafficking of cholesterol. Adv Drug Deliv Rev 49: 251–264. 11551398 10.1016/s0169-409x(01)00140-5

[36] Stahlhut M, van Deurs B (2000) Identification of filamin as a novel ligand for caveolin-1: evidence for the organization of caveolin-1-associated membrane domains by the actin cytoskeleton. Mol Biol Cell 11: 325–337. 10637311 10.1091/mbc.11.1.325 PMC14777

[37] Hailstones D, Sleer LS, Parton RG, Stanley KK (1998) Regulation of caveolin and caveolae by cholesterol in MDCK cells. J Lipid Res 39: 369–379. 9507997

[38] Hill MM, Daud NH, Aung CS, Loo D, Martin S, et al (2012) Co-regulation of cell polarization and migration by caveolar proteins PTRF/Cavin-1 and caveolin-1. PLoS ONE 7: e43041. 22912783 10.1371/journal.pone.0043041 PMC3418245

[39] Cheng ZJ, Singh RD, Holicky EL, Wheatley CL, Marks DL, et al (2010) Co-regulation of caveolar and Cdc42-dependent fluid phase endocytosis by phosphocaveolin-1. J Biol Chem 285: 15119–15125. 20228056 10.1074/jbc.M109.069427 PMC2865320

[40] Eyster CA, Higginson JD, Huebner R, Porat-Shliom N, Weigert R, et al (2009) Discovery of new cargo proteins that enter cells through clathrin-independent endocytosis. Traffic 10: 590–599. 19302270 10.1111/j.1600-0854.2009.00894.x PMC2854272

[41] Rentero C, Zech T, Quinn CM, Engelhardt K, Williamson D, et al (2008) Functional implications of plasma membrane condensation for T cell activation. PLoS ONE 3: e2262. 18509459 10.1371/journal.pone.0002262 PMC2384009

[42] Jiang H, Peterson RS, Wang W, Bartnik E, Knudson CB, et al (2002) A requirement for the CD44 cytoplasmic domain for hyaluronan binding, pericellular matrix assembly, and receptor-mediated endocytosis in COS-7 cells. J Biol Chem 277: 10531–10538. 11792695 10.1074/jbc.M108654200

[43] Hernandez-Deviez DJ, Howes MT, Laval SH, Bushby K, Hancock JF, et al (2008) Caveolin regulates endocytosis of the muscle repair protein, dysferlin. J Biol Chem 283: 6476–6488. 18096699 10.1074/jbc.M708776200

[44] Le PU, Guay G, Altschuler Y, Nabi IR (2002) Caveolin-1 is a negative regulator of caveolae-mediated endocytosis to the endoplasmic reticulum. J Biol Chem 277: 3371–3379. 11724808 10.1074/jbc.M111240200

[45] Minshall RD, Tiruppathi C, Vogel SM, Niles WD, Gilchrist A, et al (2000) Endothelial cell-surface gp60 activates vesicle formation and trafficking via G(i)-coupled Src kinase signaling pathway. J Cell Biol 150: 1057–1070. 10973995 10.1083/jcb.150.5.1057 PMC2175246

[46] Sharma P, Varma R, Sarasij RC, Ira, Gousset K, et al (2004) Nanoscale organization of multiple GPI-anchored proteins in living cell membranes. Cell 116: 577–589. 14980224 10.1016/s0092-8674(04)00167-9

[47] Bernatchez P, Sharma A, Bauer PM, Marin E, Sessa WC (2011) A noninhibitory mutant of the caveolin-1 scaffolding domain enhances eNOS-derived NO synthesis and vasodilation in mice. J Clin Invest 121: 3747–3755. 21804187 10.1172/JCI44778 PMC3163946

[48] Lajoie P, Kojic LD, Nim S, Li L, Dennis JW, et al (2009) Caveolin-1 regulation of dynamin-dependent, raft-mediated endocytosis of cholera toxin-B sub-unit occurs independently of caveolae. J Cell Mol Med 13: 3218–3225. 19438805 10.1111/j.1582-4934.2009.00732.x PMC4516479

[49] Hoffmann C, Berking A, Agerer F, Buntru A, Neske F, et al (2010) Caveolin limits membrane microdomain mobility and integrin-mediated uptake of fibronectin-binding pathogens. J Cell Sci 123: 4280–4291. 21098633 10.1242/jcs.064006

[50] Ghosh PK, Vasanji A, Murugesan G, Eppell SJ, Graham LM, et al (2002) Membrane microviscosity regulates endothelial cell motility. Nat Cell Biol 4: 894–900. 12402046 10.1038/ncb873

[51] Inder KL, Zheng YZ, Davis MJ, Moon H, Loo D, et al (2012) Expression of PTRF in PC-3 Cells modulates cholesterol dynamics and the actin cytoskeleton impacting secretion pathways. Mol Cell Proteomics 11: M111 012245. 10.1074/mcp.M111.012245 PMC3277761 22030351

[52] Breen MR, Camps M, Carvalho-Simoes F, Zorzano A, Pilch PF (2012) Cholesterol depletion in adipocytes causes caveolae collapse concomitant with proteosomal degradation of cavin-2 in a switch-like fashion. PLoS ONE 7: e34516. 22493697 10.1371/journal.pone.0034516 PMC3321009

[53] Fairn GD, Hermansson M, Somerharju P, Grinstein S (2011) Phosphatidylserine is polarized and required for proper Cdc42 localization and for development of cell polarity. Nat Cell Biol 13: 1424–1430. 21964439 10.1038/ncb2351

[54] Parat MO, Anand-Apte B, Fox PL (2003) Differential caveolin-1 polarization in endothelial cells during migration in two and three dimensions. Mol Biol Cell 14: 3156–3168. 12925753 10.1091/mbc.E02-11-0761 PMC181557

[55] Grande-Garcia A, Echarri A, de Rooij J, Alderson NB, Waterman-Storer CM, et al (2007) Caveolin-1 regulates cell polarization and directional migration through Src kinase and Rho GTPases. J Cell Biol 177: 683–694. 17517963 10.1083/jcb.200701006 PMC2064213

[56] Parton RG, Del Pozo MA (2013) Caveolae as plasma membrane sensors, protectors and organizers. Nat Rev Mol Cell Biol 14: 98–112. 23340574 10.1038/nrm3512

[57] Owen DM, Williamson DJ, Magenau A, Gaus K (2012) Sub-resolution lipid domains exist in the plasma membrane and regulate protein diffusion and distribution. Nat Commun 3: 1256. 23212385 10.1038/ncomms2273

[58] Ariotti N, Fernandez-Rojo MA, Zhou Y, Hill MM, Rodkey TL, et al (2014) Caveolae regulate the nanoscale organization of the plasma membrane to remotely control Ras signaling. J Cell Biol 204: 777–792. 24567358 10.1083/jcb.201307055 PMC3941050

[59] Bernatchez PN, Bauer PM, Yu J, Prendergast JS, He P, et al (2005) Dissecting the molecular control of endothelial NO synthase by caveolin-1 using cell-permeable peptides. Proc Natl Acad Sci U S A 102: 761–766. 15637154 10.1073/pnas.0407224102 PMC545535

[60] Nethe M, Anthony EC, Fernandez-Borja M, Dee R, Geerts D, et al (2010) Focal-adhesion targeting links caveolin-1 to a Rac1-degradation pathway. J Cell Sci 123: 1948–1958. 20460433 10.1242/jcs.062919

[61] Gonnord P, Blouin CM, Lamaze C (2012) Membrane trafficking and signaling: two sides of the same coin. Semin Cell Dev Biol 23: 154–164. 22085846 10.1016/j.semcdb.2011.11.002

[62] Couet J, Li S, Okamoto T, Ikezu T, Lisanti MP (1997) Identification of peptide and protein ligands for the caveolin-scaffolding domain. Implications for the interaction of caveolin with caveolae-associated proteins. J Biol Chem 272: 6525–6533. 9045678 10.1074/jbc.272.10.6525

[63] Kim JH, Han JM, Lee S, Kim Y, Lee TG, et al (1999) Phospholipase D1 in caveolae: regulation by protein kinase Calpha and caveolin-1. Biochemistry 38: 3763–3769. 10090765 10.1021/bi982478+

[64] Collinet C, Stoter M, Bradshaw CR, Samusik N, Rink JC, et al (2010) Systems survey of endocytosis by multiparametric image analysis. Nature 464: 243–249. 20190736 10.1038/nature08779

[65] Keller P, Toomre D, Diaz E, White J, Simons K (2001) Multicolour imaging of post-Golgi sorting and trafficking in live cells. Nat Cell Biol 3: 140–149. 11175746 10.1038/35055042

[66] Trenchi A, Gomez GA, Daniotti JL (2009) Dual acylation is required for trafficking of growth-associated protein-43 (GAP-43) to endosomal recycling compartment via an Arf6-associated endocytic vesicular pathway. Biochem J 421: 357–369. 19442238 10.1042/BJ20090484

[67] Thomsen P, Roepstorff K, Stahlhut M, van Deurs B (2002) Caveolae are highly immobile plasma membrane microdomains, which are not involved in constitutive endocytic trafficking. Mol Biol Cell 13: 238–250. 11809836 10.1091/mbc.01-06-0317 PMC65085

[68] Yguerabide J, Schmidt JA, Yguerabide EE (1982) Lateral mobility in membranes as detected by fluorescence recovery after photobleaching. Biophys J 40: 69–75. 7139035 10.1016/S0006-3495(82)84459-7 PMC1328974

[69] Schneider M, Barozzi S, Testa I, Faretta M, Diaspro A (2005) Two-photon activation and excitation properties of PA-GFP in the 720–920-nm region. Biophys J 89: 1346–1352. 15908572 10.1529/biophysj.104.054502 PMC1366619

[70] Rahkila P, Alakangas A, Vaananen K, Metsikko K (1996) Transport pathway, maturation, and targetting of the vesicular stomatitis virus glycoprotein in skeletal muscle fibers. J Cell Sci 109 (Pt 6) 1585–1596. 8799845 10.1242/jcs.109.6.1585

