# Supplementary material for: Endocytic Crosstalk: Cavins, Caveolins, and Caveolae Regulate Clathrin-Independent Endocytosis
Source: PLoS Biol. 2014 Apr 8;12(4):e1001832. doi: 10.1371/journal.pbio.1001832 (PMC3979662; doi:10.1371/journal.pbio.1001832)
Supplement: Text S1 — Supplementary experimental procedures (DOCX) [file pbio.1001832.s001.docx]

**Supplementary Experimental Procedures**

**Western blotting**

Whole cell lysates were prepared in TNE buffer (20 mM Tris, 150 mM NaCl, 5 mM EDTA, protease and phosphatase inhibitors) and protein samples were run on a 0.1% SDS, 12% polyacrylamide gel at 180 Volts in 1X SDS-PAGE running buffer (25 mM Tris, 250 mM Glycine, 0.1% SDS). Resolved samples were transferred from SDS-PAGE gels to PVDF membrane (Whatman Schleicher Schuell Bioscience), using 1X SDS-PAGE transfer buffer (48 mM Tris, 39 mM Glycine, 20% methanol). Following transfer, membranes were washed for 15 min (2 times) in blocking solution (5% w/v skim milk powder, 0.1% Tween20 in TBS, TBST) before incubating them in primary antibody. Membranes were washed for 10 min (3 times) with TBST between primary and secondary antibody incubations.

**FRAP: determination of mobile fraction and diffusion rate**

The average fluorescence (F(t)) intensity of the bleached area (bl) and a reference (ref) and non-bleached area before and after the bleaching process where quantitated using Image J. Recovery plots were analyzed as described previously [[1](#_ENREF_1)] using the following equations:

$Q\left( t \right)=\frac{F_{bl}(t)}{F_{ref}(t)}$ … (1) and,

Relative fluorescence recovery =$\frac{Q\left( t \right)-Q\left( t_{0} \right)}{Q\left( t_{pre} \right)-Q\left( t_{0} \right)}$ … (2)

In the last equation $Q\left( t_{0} \right)$ and $Q\left( t_{pre} \right)$ are values of $Q\left( t \right)$ obtained in the first frame after bleaching $\left( t_{0} \right)$ and the average $Q\left( t \right)$ for frames acquired before the bleaching process $\left( t_{pre} \right)$. To analyze GPI-YFP dynamics, relative fluorescence recovery were then fitted to the double exponential equation:

Relative Fluorescence Recovery$=Mf\cdot\left[ f_{fast}\cdot(1-e^{\frac{ln2\cdot t}{t_{\frac{1}{2}}^{fast}}}) \right]+Mf\cdot\left[ f_{slow}\cdot(1-e^{\frac{ln2\cdot t}{t_{\frac{1}{2}}^{slow}}}) \right]$ (3)

where is the mobile fraction, $f_{fast}$ and $f_{slow}$ are weighting factors for fast and slow mobile components, $t_{1/2}^{fast}$ and $t_{1/2}^{slow}$ their respective half times and t is time in seconds. A numerical solution of the above equation for Fluorescence Recovery = 0.5 was applied to obtain the global half-time of GPI fluorescence recovery. Diffusion coefficients calculations were performed as described before using the following equation [[2](#_ENREF_2),[3](#_ENREF_3)].

$D=\frac{0.88 * \omega^{2}}{4 * t_{1/2}}$ (4), where ω is the radius of the bleached disc.

For CAV1-YFP dynamics, relative fluorescence recovery for FRAP curves were fitted to the following equation as it was described before [[4](#_ENREF_4),[5](#_ENREF_5)]:

Relative Fluorescence Recovery$=\frac{Mf*\left( \frac{t}{t_{1/2}} \right)}{1+\left( \frac{t}{t_{1/2}} \right)}$ (5)

$t_{1/2}$ values were obtained for experiments performed using different bleach area sizes. Linear regression of $t_{1/2}$ values against bleach area disc plots were used to calculate diffusion coefficient for Caveolin using equation 4.

**Muscle fiber isolation and analysis of endocytic activity**

Muscle fibers were isolated from the flexor digitorum brevis (FDB) of adult Cavin-1-/- and WT littermate control mice. FDB muscle was incubated in Worthington’s type II collagenase (700 U/ml) prepared in dissociation medium (DMEM, 2 mM L-glutamine, 100 μg/ml streptomycin and 100 U/ml penicillin). Fibers were resuspended in growth media (dissociation media supplemented with 10% FBS). 3 cm cell culture dishes were coated with Matrigel (Becton Dickinson), the fiber suspension was spread onto the dish and fibers allowed to adhere for 2 h or overnight at 37 °C. For performing internalization assays, fibers were incubated with endocytic markers at 37 °C for the desired amount of time. To remove any surface bound markers acid striping was performed 20 sec (2 times) with 0.5 M Glycine, pH 2.2 before fixing the fibers in 4% paraformaldehyde. Immunofluorescence was performed as previously described [[6](#_ENREF_6)].

1. Trenchi A, Gomez GA, Daniotti JL (2009) Dual acylation is required for trafficking of growth-associated protein-43 (GAP-43) to endosomal recycling compartment via an Arf6-associated endocytic vesicular pathway. Biochem J 421: 357-369.

2. Axelrod D, Koppel DE, Schlessinger J, Elson E, Webb WW (1976) Mobility measurement by analysis of fluorescence photobleaching recovery kinetics. Biophys J 16: 1055-1069.

3. Hoffmann C, Berking A, Agerer F, Buntru A, Neske F, et al. (2010) Caveolin limits membrane microdomain mobility and integrin-mediated uptake of fibronectin-binding pathogens. J Cell Sci 123: 4280-4291.

4. Thomsen P, Roepstorff K, Stahlhut M, van Deurs B (2002) Caveolae are highly immobile plasma membrane microdomains, which are not involved in constitutive endocytic trafficking. Mol Biol Cell 13: 238-250.

5. Yguerabide J, Schmidt JA, Yguerabide EE (1982) Lateral mobility in membranes as detected by fluorescence recovery after photobleaching. Biophys J 40: 69-75.

6. Rahkila P, Alakangas A, Vaananen K, Metsikko K (1996) Transport pathway, maturation, and targetting of the vesicular stomatitis virus glycoprotein in skeletal muscle fibers. J Cell Sci 109 ( Pt 6): 1585-1596.
