# Supplementary figures and images for: Endocytic Crosstalk: Cavins, Caveolins, and Caveolae Regulate Clathrin-Independent Endocytosis
Source: PLoS Biol. 2014 Apr 8;12(4):e1001832. doi: 10.1371/journal.pbio.1001832 (PMC3979662; doi:10.1371/journal.pbio.1001832)

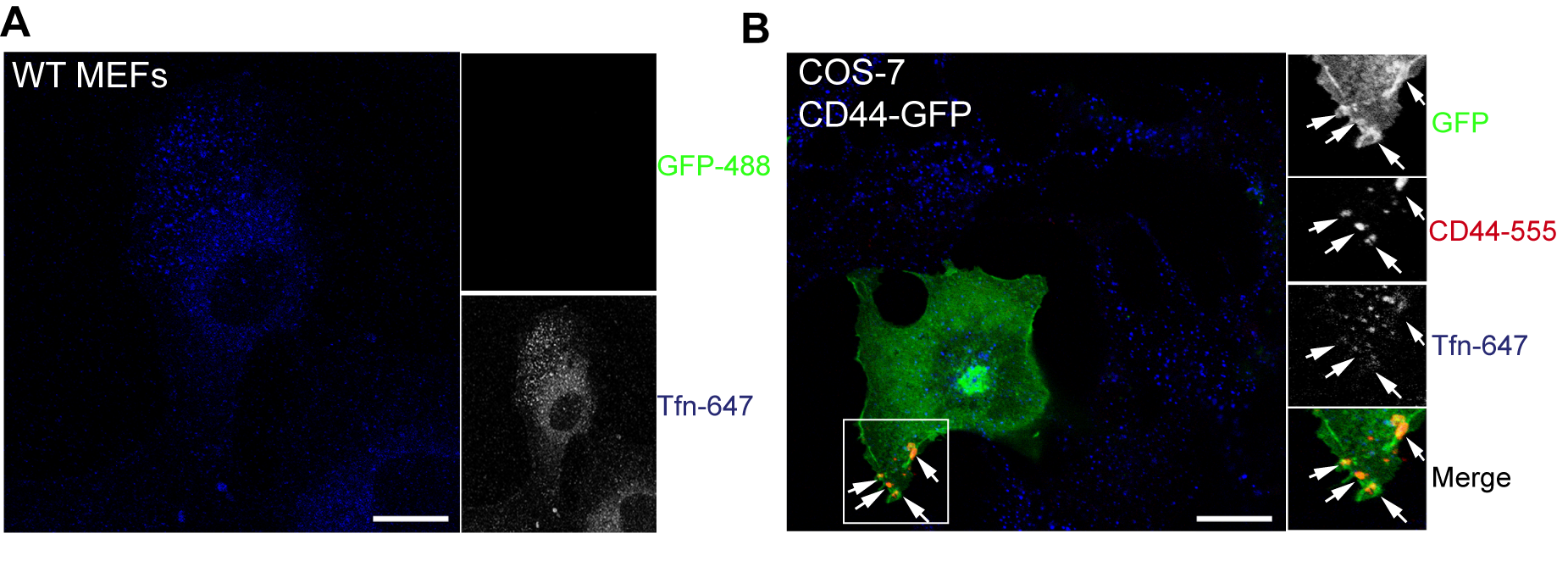

Supplement: Figure S1 — CD44 mAb as specific cargo of the CLIC/GEEC pathway. (A) Internalization assay was performed in WT MEFs with anti-GFP mAb and Tfn-647 for 2 min at 37°C. Cells were acid washed prior to fixation and for labeling internalized GFP mAb secondary AF-488 antibody was used. (B) COS-7 cells were transiently transfected with CD44-GFP and internalization assay was performed with anti-CD44 mAb and Tfn-647 for 2 min at 37°C. Cells were acid washed prior to fixation and internalized anti-CD44 mAb was labeled with AF-555 secondary antibody. Arrows denote CD44 labeled puncta. Scale bar: 10 µm. (TIF) [file pbio.1001832.s002.tif]

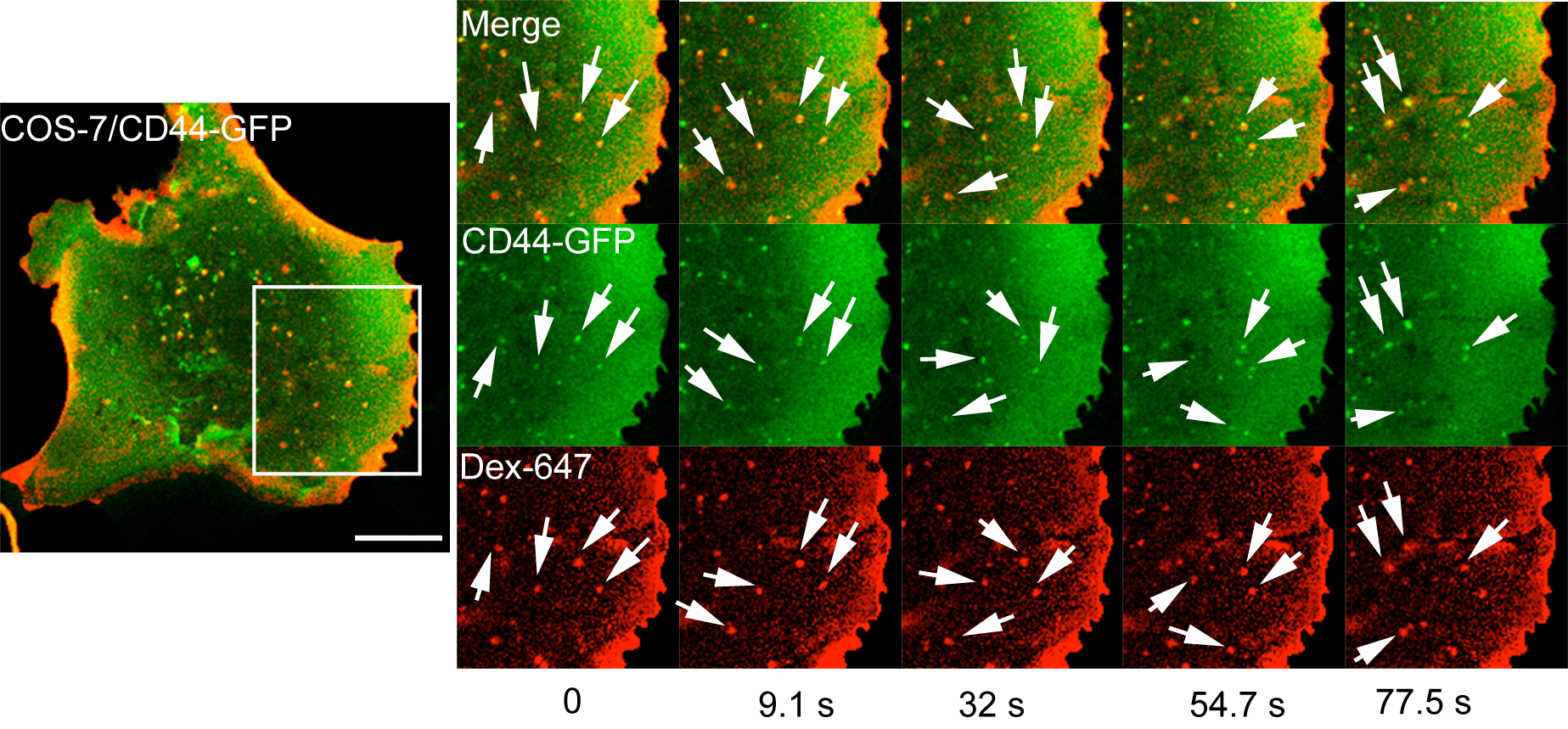

Supplement: Figure S2 — CD44-GFP labeled vesicles co-localize with internalized Dex-647. COS-7 cells transfected with CD44-GFP were imaged live at 37°C in presence of Dex-647 (2 mg/ml). Time-lapse covers a period of 7 min and images from the selected frames of the movie (Movie S1) are shown. Scale bar: 10 µm. (TIF) [file pbio.1001832.s003.tif]

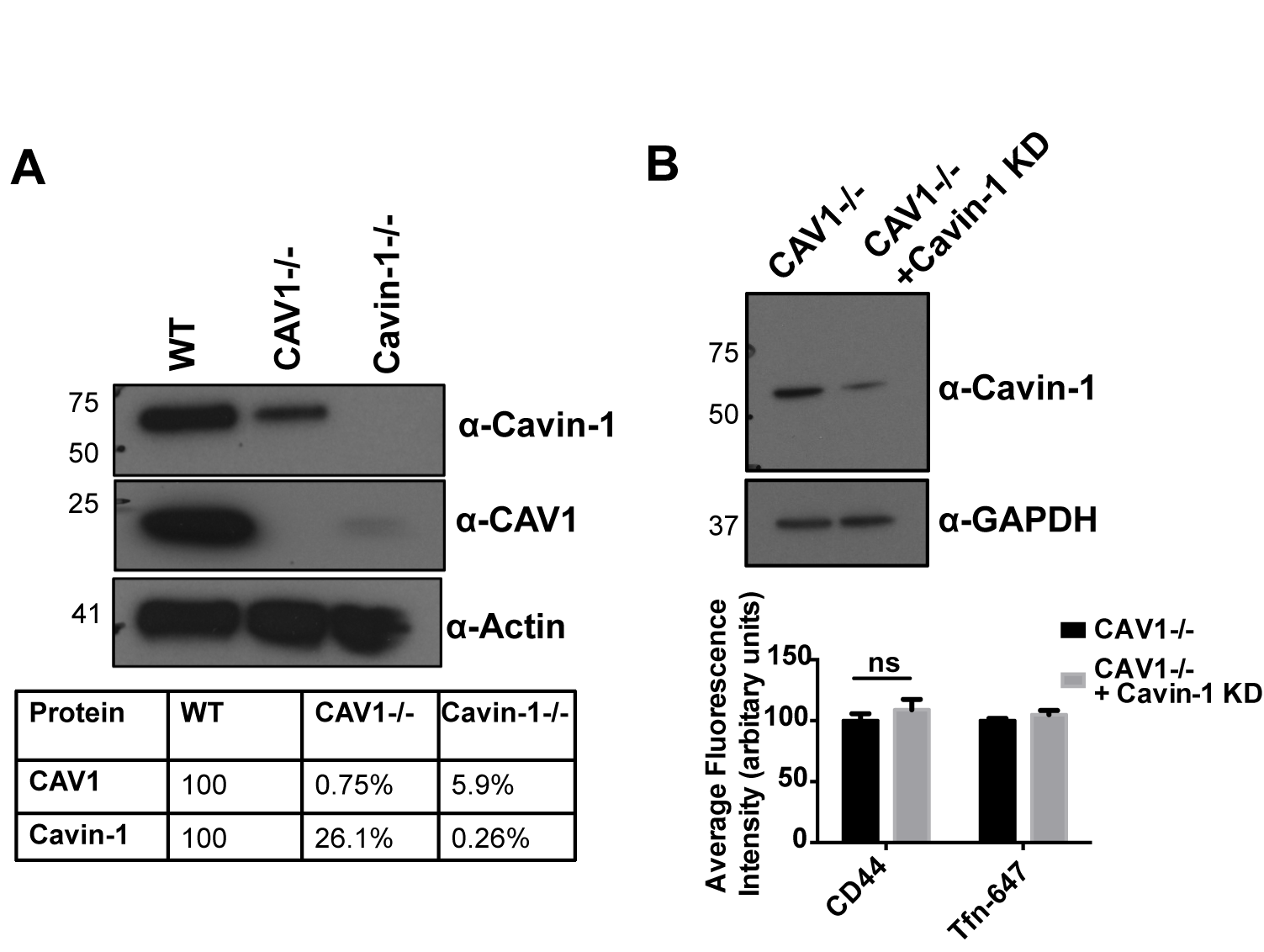

Supplement: Figure S3 — Protein levels of caveolar components in WT, CAV1−/−, and Cavin-1−/− MEFs. (A) Whole cell lysates from WT, CAV1−/− and Cavin-1−/− MEFs were immunoblotted with CAV1 and Cavin-1 primary antibodies followed by secondary HRP-conjugated antibodies. Actin was used as a loading control. For quantitative analysis of protein levels, Densitometric analysis of band intensities was performed. (B) Whole cell lysates from CAV1−/− and CAV1−/− expressing Cavin-1-specific siRNA were immunoblotted with Cavin-1 primary antibody followed by secondary HRP-conjugated antibodies. GAPDH was used as a loading control. A representative immunoblot is shown. The same set of transfected cells growing on coverslips were subjected to internalization assays with anti-CD44 mAb and Tfn-647 for 2 min at 37°C. Cells were acid washed prior to fixation. Internalized CD44 mAb was detected with an AF-555-labeled secondary antibody. The bar graph represents the quantification of internalized markers. Data represent mean ± SEM of three independent experiments. (TIF) [file pbio.1001832.s004.tif]

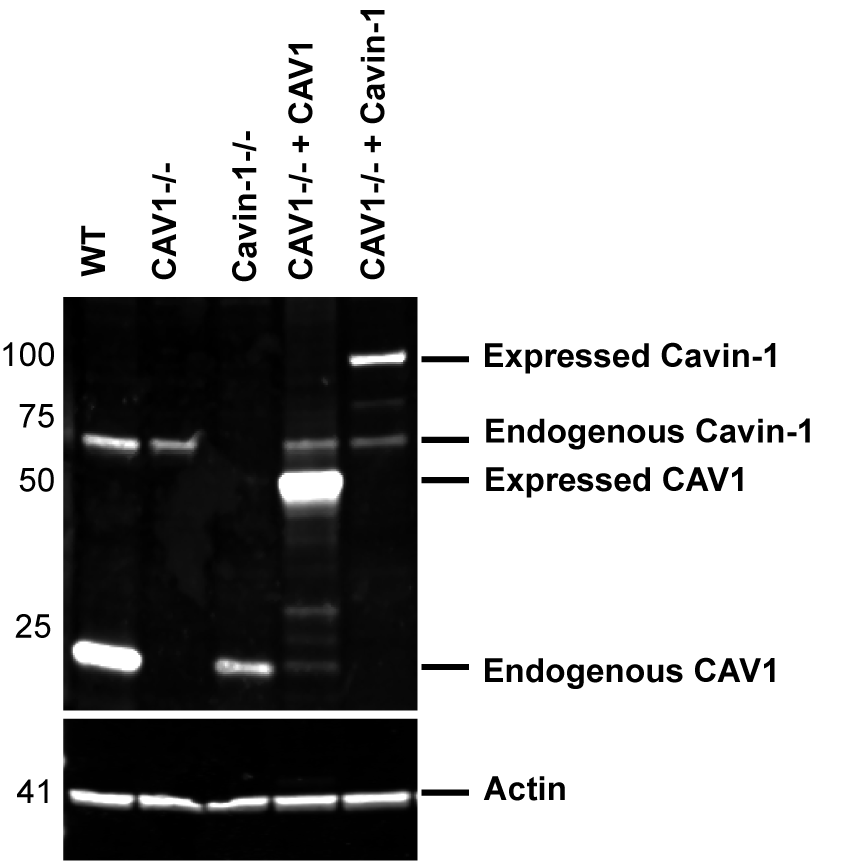

Supplement: Figure S4 — Reconstitution of CAV1 and Cavin-1 in CAV1−/− MEFs. Whole cell lysates were prepared from WT, CAV1−/−, Cavin-1−/−, and CAV1−/− MEFs transiently transfected with CAV1-GFP and Cavin-1-GFP respectively. Lysates were immunoblotted with CAV1 and Cavin-1 primary antibodies followed by secondary fluorescent (Odyssey) antibodies. Actin was used as a loading control, and for detection the Licor Odyssey infrared imaging system was used. (TIF) [file pbio.1001832.s005.tif]

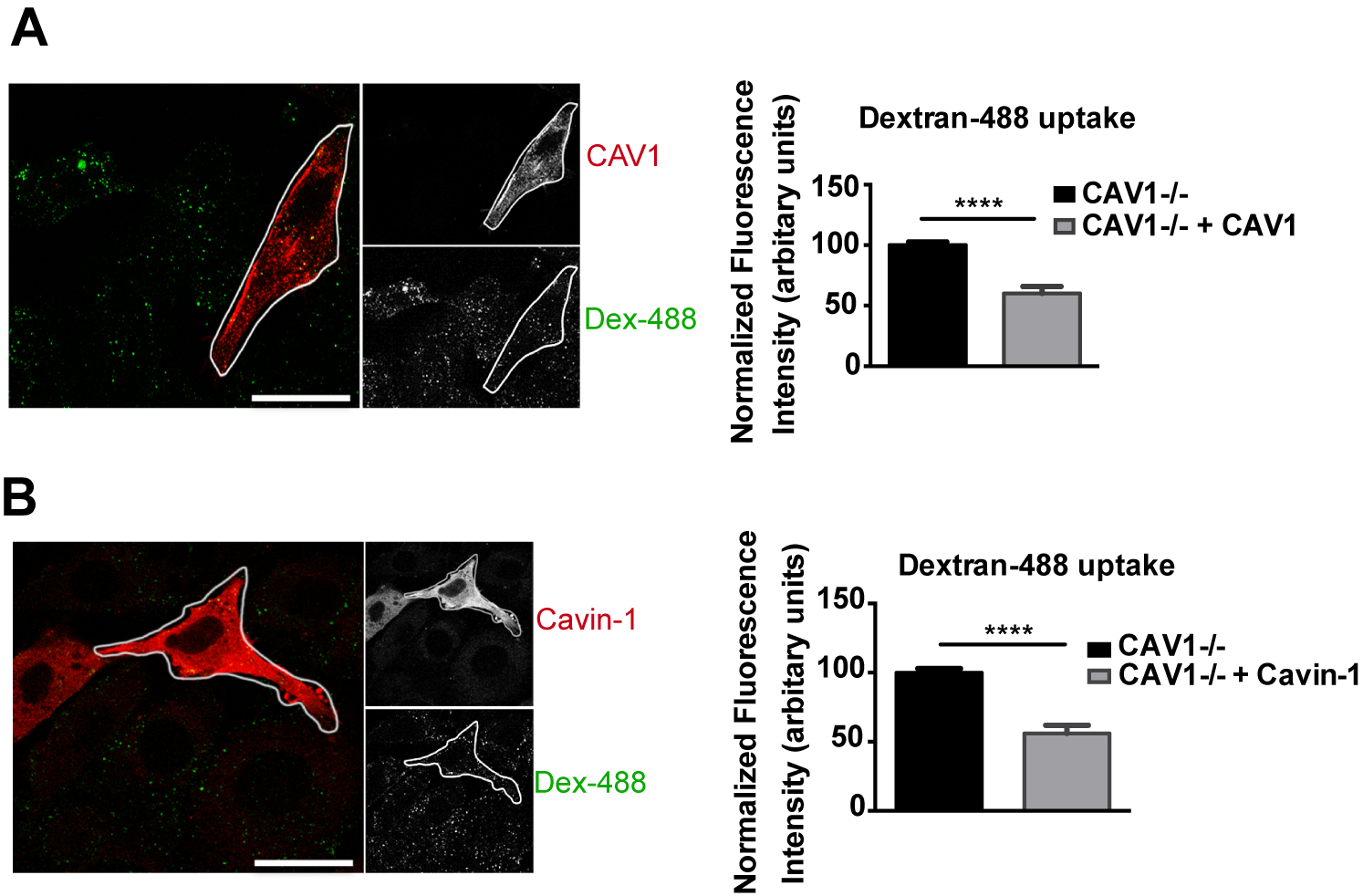

Supplement: Figure S5 — Inhibition of Dex-488 uptake by CAV1 and Cavin-1 in CAV1−/− MEFs. (A) CAV1−/− MEFs were transiently transfected with CAV1-YFP and (B) with Cavin-1-GFP respectively. Internalization assay was performed with Dex-488 for 5 min at 37°C. 40–50 cells from each transfection from (A, B) were quantified for normalized fluorescent intensity of internalized Dex-488. Untransfected CAV1−/− MEFs represent control. In (A,B) data represent mean ± SEM of three independent experiments. ****p<0.0001 (two-tailed t-test). Scale bar: 10 µm. (TIF) [file pbio.1001832.s006.tif]

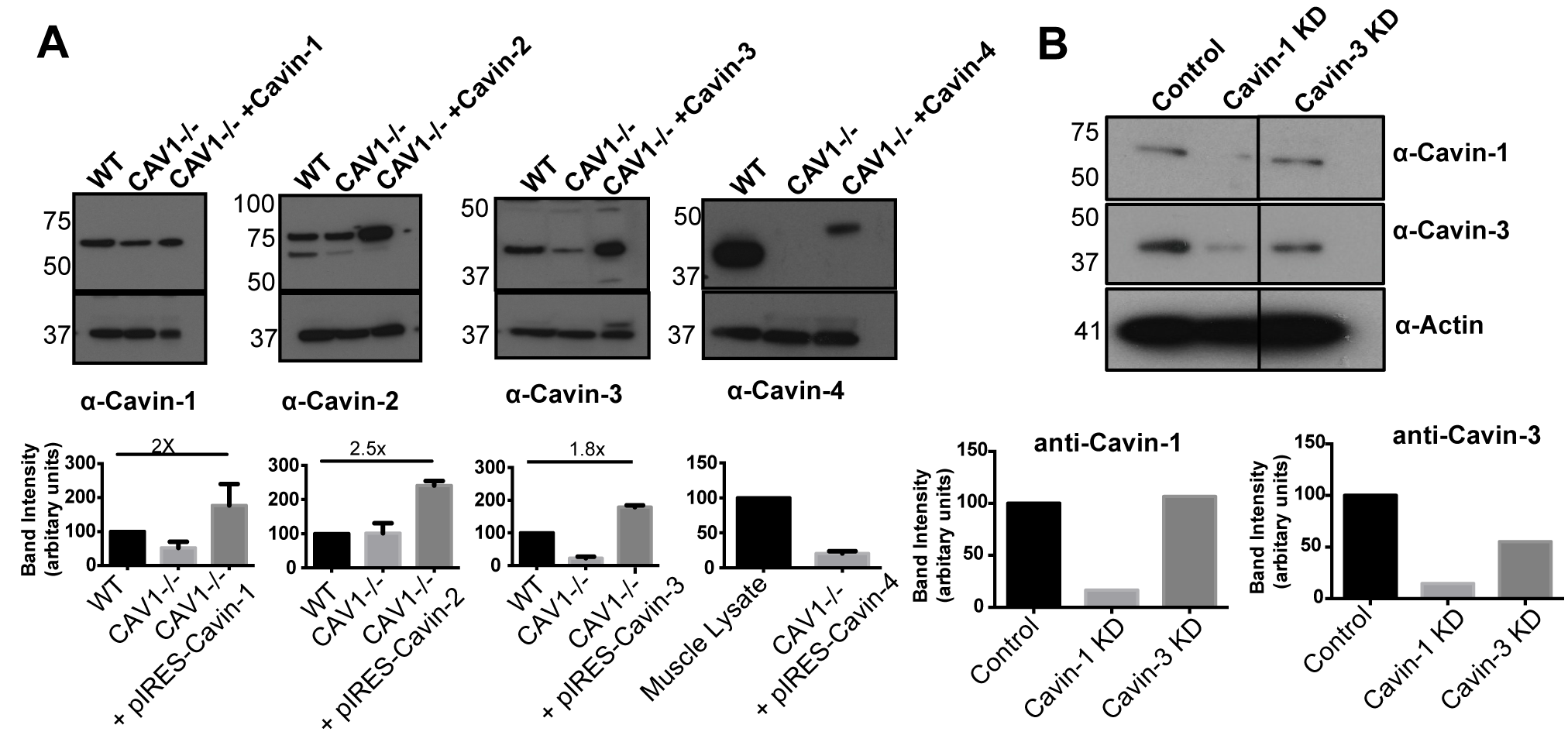

Supplement: Figure S6 — Cavin-mediated inhibition of the CLIC/GEEC pathway. (A) CAV1−/− MEFs were transiently transfected with pIRES-Cavin-1, pIRES-Cavin-2, pIRES-Cavin-3 and pIRES-Cavin-4 respectively. Whole cell lysates from above transfected CAV1−/− MEFs, untransfected WT MEFs, untransfected CAV1−/− MEFs, and muscle tissue were immunoblotted with respective cavin primary antibodies followed by secondary HRP-conjugated antibodies. Lysates from untransfected CAV1−/− MEFs and WT were used as a control for Cavin-1–3 endogenous expression levels, while muscle lysates were used specifically as control for Cavin-4 endogenous expression. GAPDH was used as loading control. A representative Western blot is shown. The bar graphs represent densitometric analysis results of respective cavin protein levels (mean ± SEM; from three independent experiments) normalized to the values obtained in WT lysates. (B) 3T3-L1 cells were transiently transfected with siRNA directed to Cavin-1 and Cavin-3 respectively. 48 h post transfection cells lysates were immunoblotted with respective Cavin-1 and Cavin-3 primary antibodies followed by secondary HRP-conjugated antibodies. A representative Western blot is shown and lanes for control, Cavin-1 and Cavin-3 are cropped sections of the same film. The bar graph represents quantitation of Cavin-1 and Cavin-3 protein levels normalized to control levels, measured by densitometry. Actin was used as a loading control. (TIF) [file pbio.1001832.s007.tif]

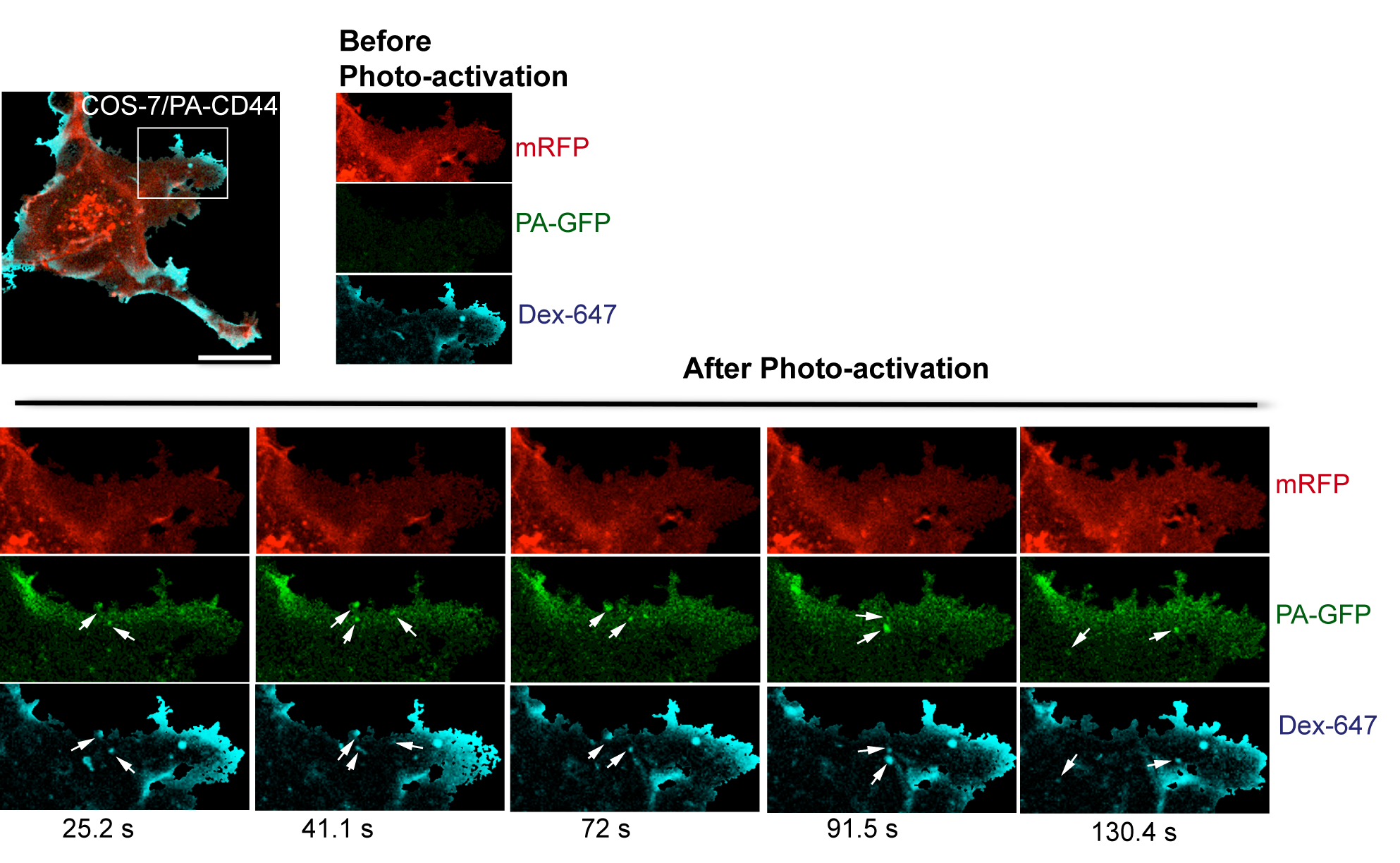

Supplement: Figure S7 — Photo-activated CD44 (PA-CD44) labeled endocytic carriers co-localize with internalized dextran. COS-7 cells were transfected with PA-CD44 and a selected ROI at PM was photo-activated and imaged at 37°C in presence of Dex-647 (2 mg/ml). Time-lapse covers a period of 7 min and images from the selected frames of the movie (Movie S2) are shown. Scale bar: 10 µm. (TIF) [file pbio.1001832.s008.tif]

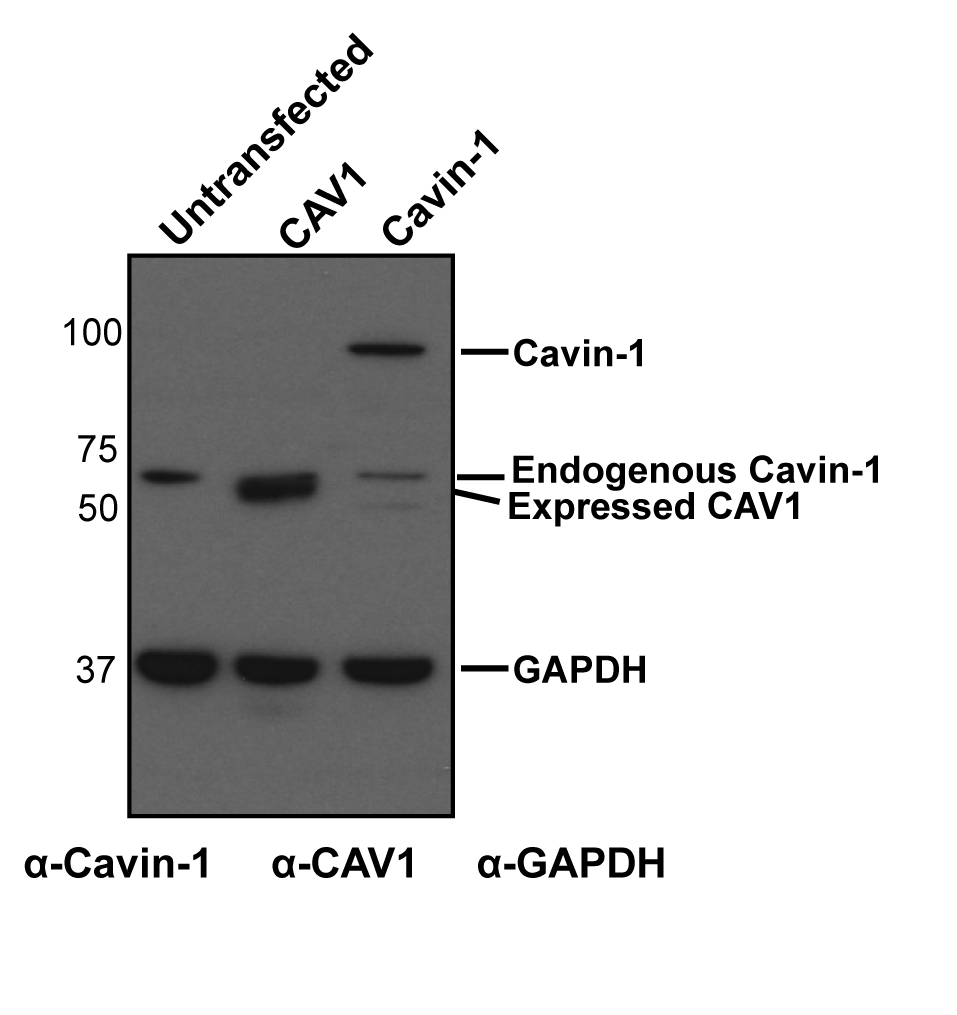

Supplement: Figure S8 — CAV1-YFP and Cavin-1-GFP expression in CAV1−/− MEFs. CAV1−/− MEFs were transiently transfected with CAV1-YFP and Cavin-1-GFP, respectively. Whole cell lysates were immunoblotted with CAV1 and Cavin-1 primary antibodies followed by secondary HRP-conjugated antibodies. GAPDH expression was used as a loading control. (TIF) [file pbio.1001832.s009.tif]

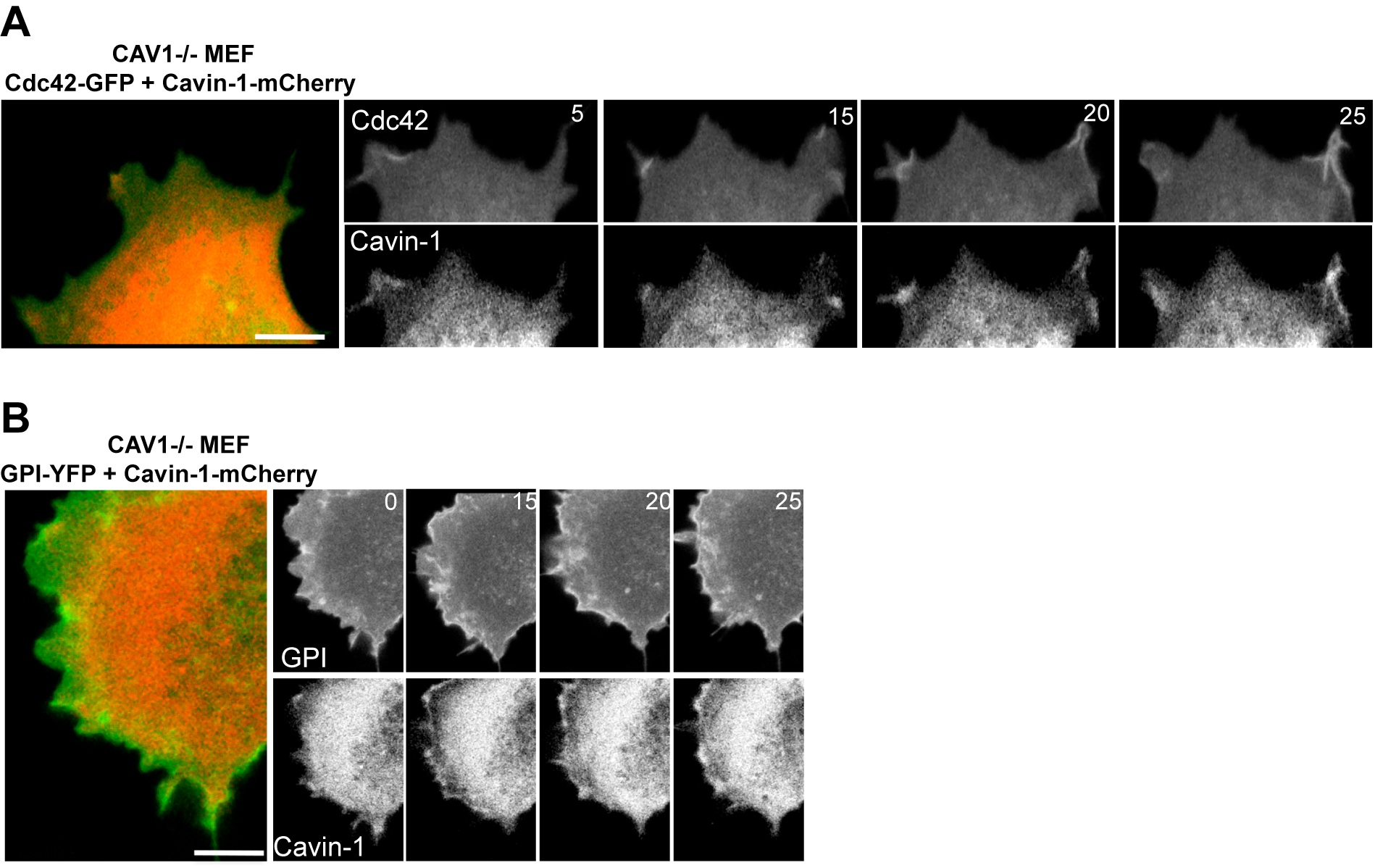

Supplement: Figure S9 — Cavin-1 co-localize with Cdc42 and GPI-AP at PM ruffles. CAV1−/− MEFs were co-transfected with (A) Cdc42-GFP and Cavin-1-mCherry and with (B) GPI-YFP and Cavin-1-mCherry respectively, and cells were imaged live at 37°C. Time-lapse covers a period of 18 min and images from the selected frames of the movie are shown. Scale bar: 10 µm. (TIF) [file pbio.1001832.s010.tif]

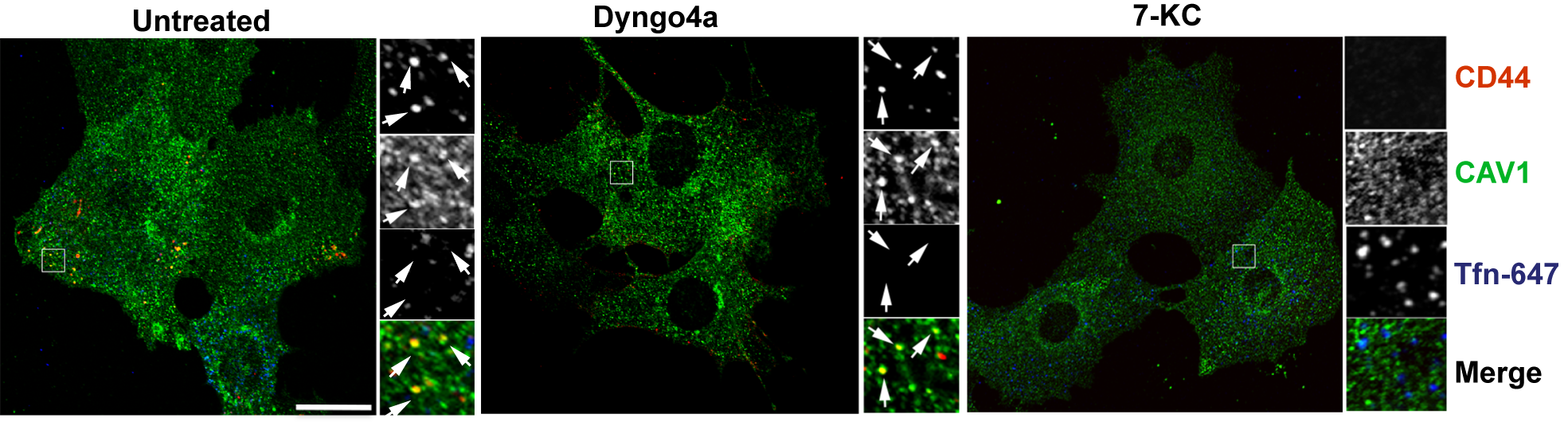

Supplement: Figure S10 — Noncaveolar CAV1 is internalized via the CLIC/GEEC pathway. Cavin-1−/−MEFs were either left untreated or treated with 60 µM Dyngo4a and 30 µM 7-KC respectively for 30 min prior to performing internalization assay with CD44 mAb and Tfn-647 for 2 min at 37°C. Endogenous CAV1 was labeled with respective primary antibodies followed by AF-488 secondary antibody labeling, and for internalized anti-CD44 mAb labeling AF-555 secondary antibody was used. Scale bar: 10 µm. (TIF) [file pbio.1001832.s011.tif]

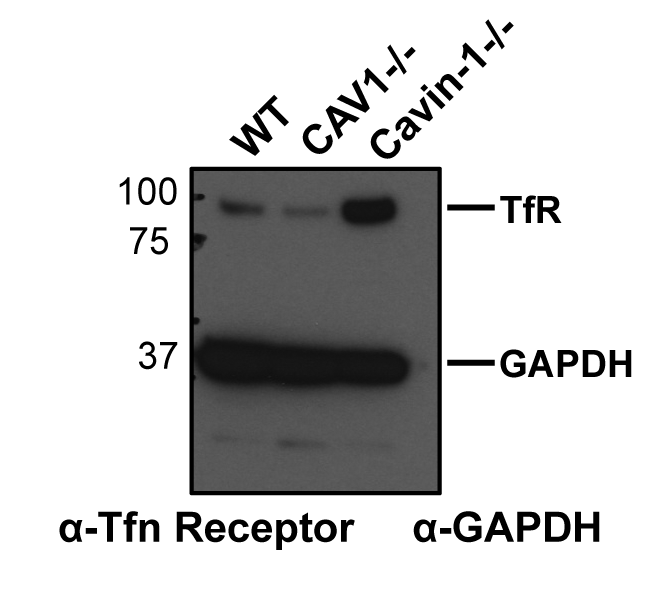

Supplement: Figure S11 — Transferrin receptor levels in CAV1−/− and Cavin-1−/− MEFs. Whole cell lysates from WT, CAV1−/− and Cavin-1−/− MEFs were immunoblotted with primary anti-transferrin receptor antibody followed by secondary HRP-conjugated antibodies. GAPDH was used as a loading control. (TIF) [file pbio.1001832.s012.tif]

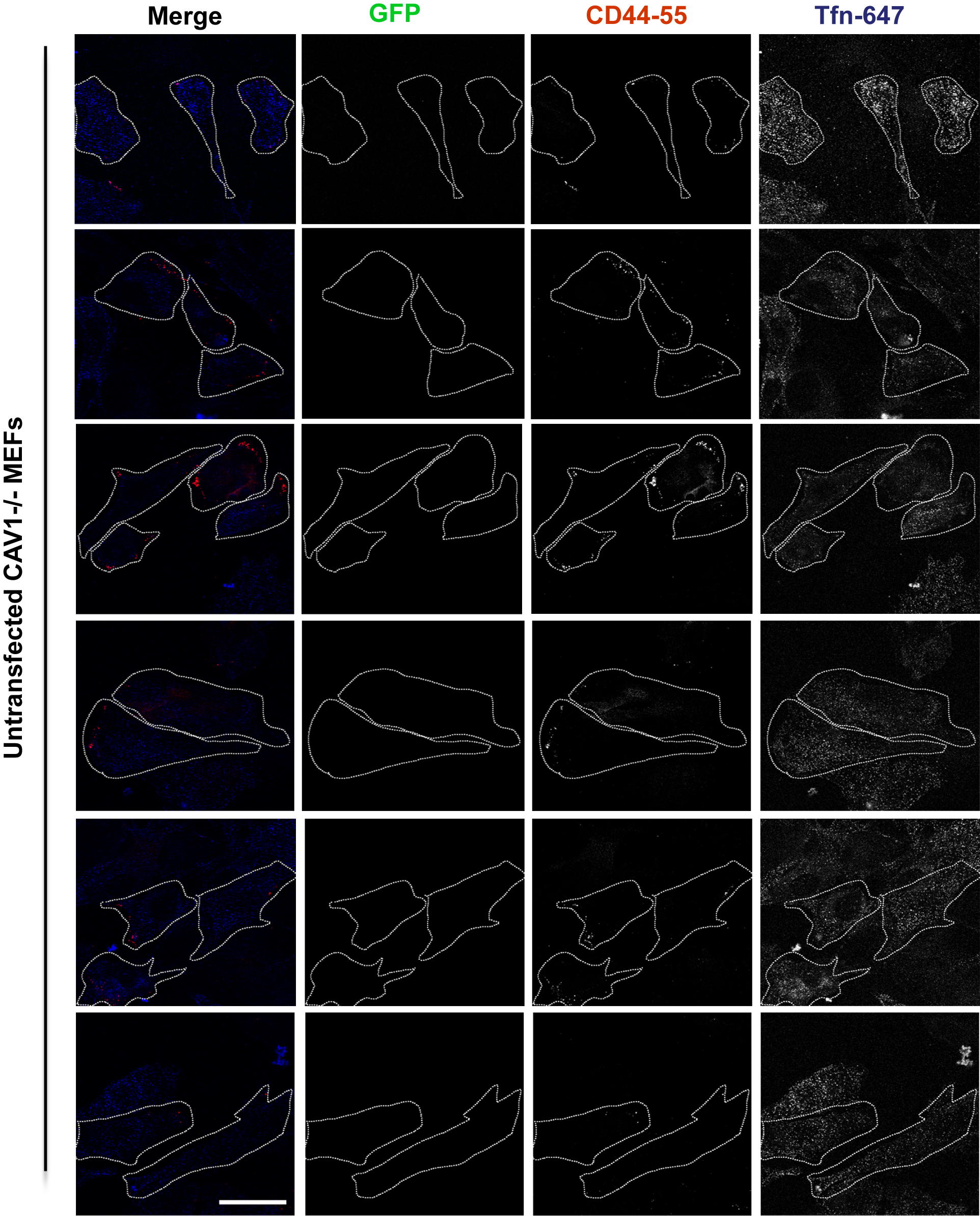

Supplement: Figure S12 — Anti-CD44 mAb and Tfn-647 internalization in CAV1−/− MEFs. CAV1−/− MEFs were incubated with anti-CD44 mAb and Tfn-647 for 2 min at 37°C. Cells were the placed on ice and acid washed before fixation. Internalized anti-CD44 mAb was labeled with AF-555 secondary antibody. Scale bar 10 µm. (TIF) [file pbio.1001832.s013.tif]

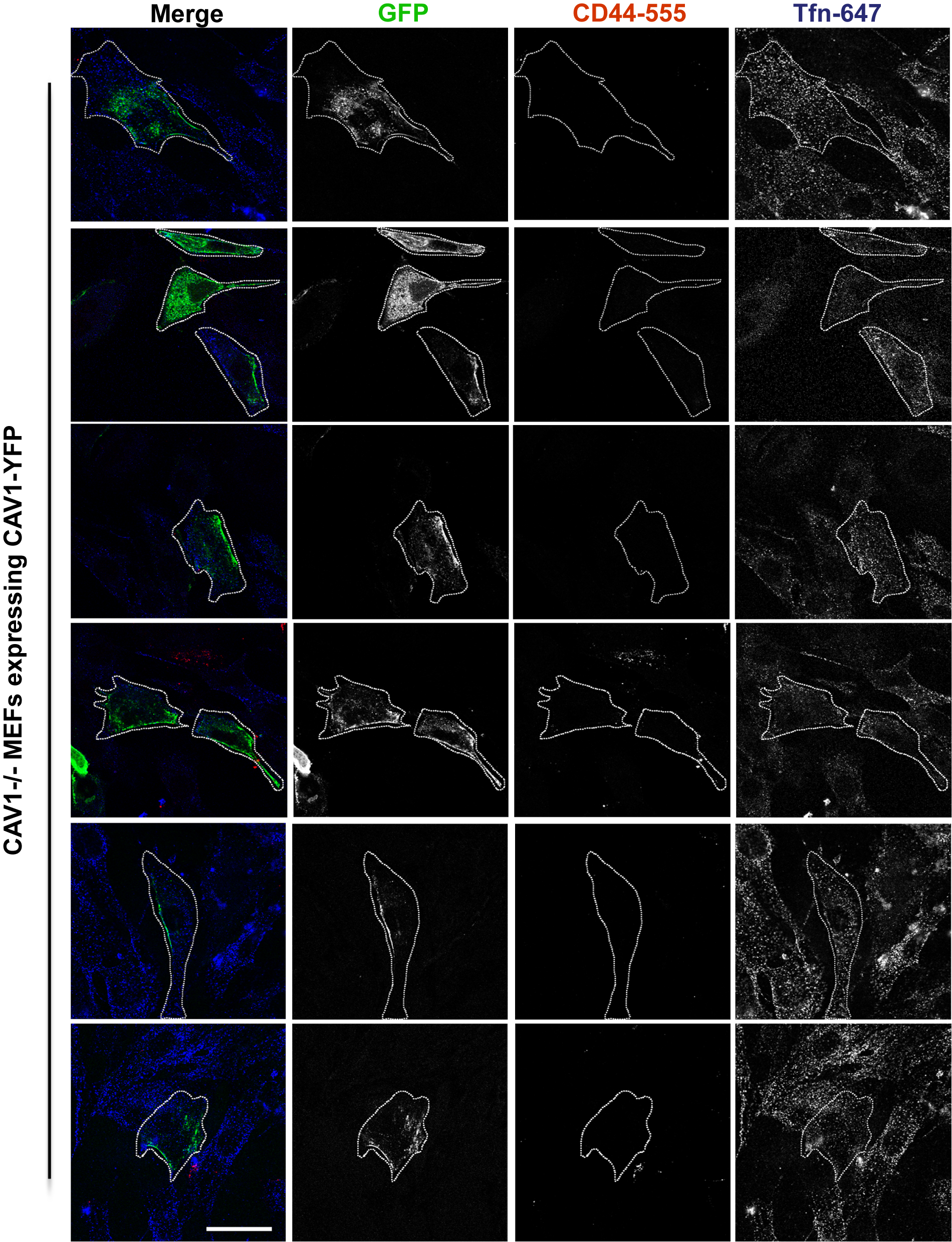

Supplement: Figure S13 — Anti-CD44 mAb and Tfn-647 internalization in CAV1-YFP expressing CAV1−/− cells. CAV1−/− MEFs were transiently transfected with Cavin-1-GFP. Post-transfection cells were incubated with anti-CD44 mAb and Tfn-647 for 2 min at 37°C. Cells were then placed on ice and acid washed before fixation. Internalized anti-CD44 mAb was labeled with AF-555 secondary antibody. Scale bar 10 µm. (TIF) [file pbio.1001832.s014.tif]

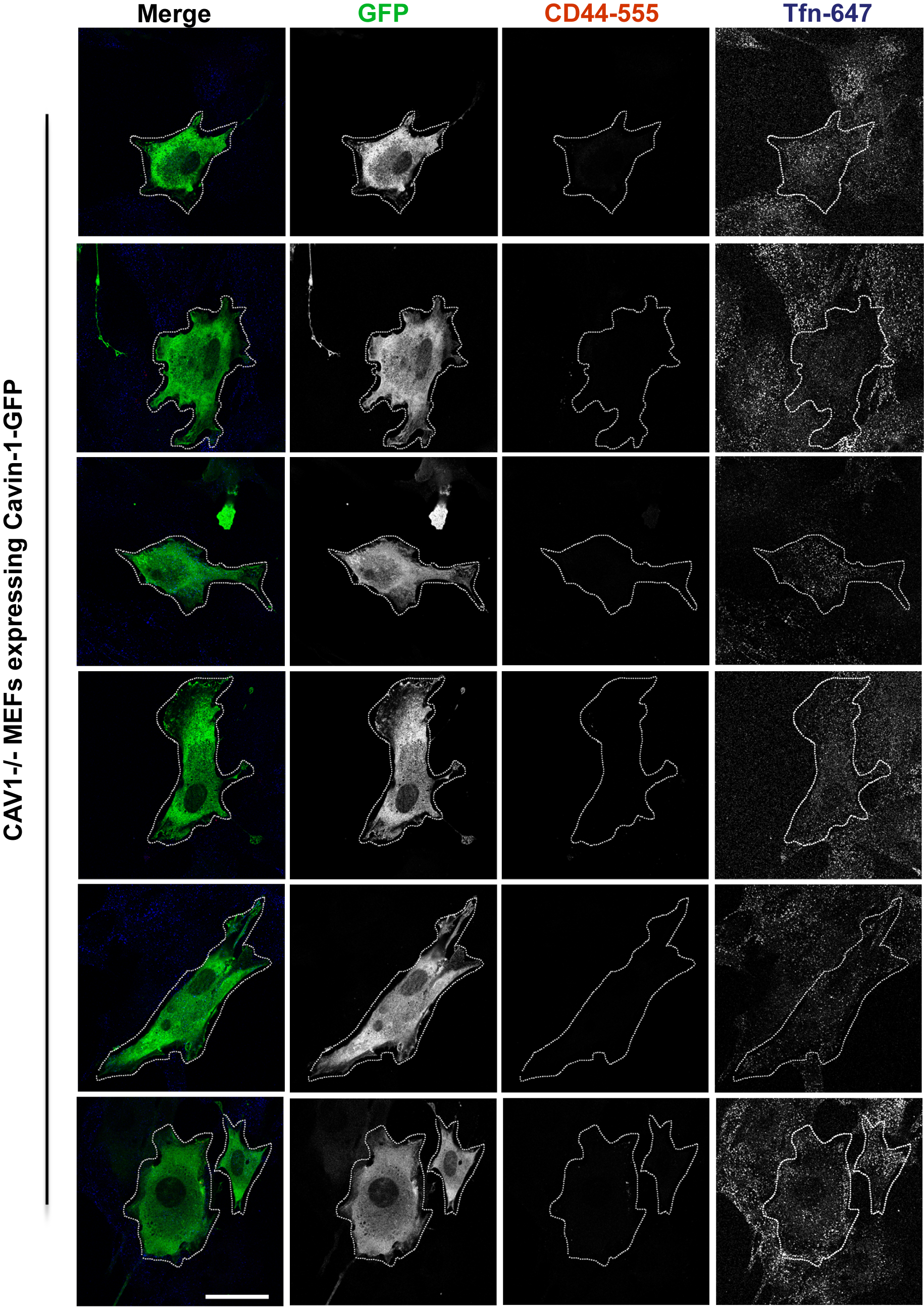

Supplement: Figure S14 — Anti-CD44 mAb and Tfn-647 internalization in Cavin-1-GFP expressing CAV1−/− cells. CAV1−/− MEFs were transiently transfected with Cavin-1-GFP. Post-transfection cells were incubated with anti-CD44 mAb and Tfn-647 for 2 min at 37°C. Cells were then placed on ice and acid washed before fixation. Internalized anti-CD44 mAb was labeled with AF-555 secondary antibody. Scale bar: 10 µm. (TIF) [file pbio.1001832.s015.tif]
